# Transport of secondary metabolites in plants: Mechanistic insights and transporter engineering for crop improvement

**DOI:** 10.1016/j.xplc.2025.101536

**Published:** 2025-09-25

**Authors:** Chunsheng Xiao, Gaofeng Zhou, Tianhua He, Chengdao Li

**Affiliations:** 1 Western Crop Genetic Alliance, Murdoch University, Perth, WA 6150, Australia; 2 State Agricultural Biotechnology Centre (SABC), College of Science, Health, Engineering and Education, Murdoch University, Perth, WA 6150, Australia; 3 Department of Primary Industry and Regional Development, Government of Western Australia, South Perth, WA 6155, Australia

**Keywords:** secondary metabolites, transporters, long-distance transport, genetic manipulation, crop improvement

## Abstract

Secondary metabolites (SMs) are essential for plant survival and adaptation, playing multiple roles in ecological interactions such as defense and stress tolerance. Specialized transporters relocate SMs from their synthesis sites to defense tissues or storage organs. The spatiotemporal distribution of defense-related SMs is a key determinant of plant fitness. However, the accumulation of anti-nutritional SMs in crop seeds or fruits can pose health risks to humans and livestock. Recent advances have revealed the critical role of SM transporters in optimizing metabolite allocation. This review examines the transport mechanisms of both defense and anti-nutritional SMs, with a particular focus on long-distance transporters that regulate source–sink dynamics and their potential applications in agricultural biotechnology. We highlight innovative strategies to manipulate transporter activity, ranging from multi-omics integration to precision engineering, and discuss how these approaches can be used to design crops with enhanced defense capacity, increased levels of beneficial compounds, and improved palatability of seeds and fruits. Finally, we outline emerging technologies and conceptual frameworks for discovering and characterizing long-distance SM transporters for crop improvement. Transporter-focused strategies offer promising solutions to global agricultural challenges and provide new opportunities for advancing crop improvement in the context of global food security.

## Teaser

Secondary metabolites (SMs) are pivotal to plant defense, adaptation, and biological interactions. This review summarizes the current understanding of SM transport mechanisms and the associated transporters in plants and proposes innovative frameworks to modulate transporter activity for crop improvement.

## Introduction

Plants produce specialized secondary metabolites (SMs) that enable them to defend against stress and adapt to challenging environments (Isah, 2019). Defense-related SMs, including alkaloids, glucosinolates (GSLs), flavonoids, terpenoids, and phenolic compounds, play vital roles in plant responses to abiotic and biotic stresses (Steppuhn et al., 2004; Freeman and Beattie, 2008; Clay et al., 2009). In addition to their defensive functions, these metabolites exhibit significant bioactive properties and hold considerable pharmaceutical potential. They are widely used in medicines, phytomedicines, nutraceuticals, fragrances, dyes, flavorings, and pesticides (Anokhina et al., 2008; He and Giusti, 2010; Riaz et al., 2023). However, certain SMs in fruits and grains can be bitter or toxic, exhibiting teratogenic or anticholinergic effects that render them unsuitable for human or livestock consumption (Wink, 2010; Wang et al., 2024).

In crop breeding, a major challenge is balancing two objectives: maintaining or enhancing stress tolerance through the accumulation of valuable defense-related SMs while improving the quality of edible plant parts by reducing anti-nutritional SMs (Dwivedi et al., 2021; Qin et al., 2023). A deeper understanding of SM metabolism—particularly the transport pathways that determine their spatiotemporal distribution—is crucial for reconciling these goals. Recent advances in SM transport biology have created new opportunities for the precise engineering of SM allocation, paving the way for crop improvement (Nogia and Pati, 2021; Bai et al., 2024).

Most defense-related and anti-nutritional SMs are synthesized in source organs (e.g., leaves, stems, pods, and roots) and subsequently transported to sink organs (e.g., rhizomes, flowers, fruits, and seeds), where they perform protective and other biological functions (Nour-Eldin and Halkier, 2009; Verma et al., 2012; Jørgensen et al., 2015; Otterbach et al., 2019). This spatial separation requires tightly regulated transport mechanisms to ensure the precise allocation of SMs to target cells and tissues. Transport occurs at multiple levels—including intracellular, intercellular, and long-distance organ-to-organ movement (Shitan and Yazaki, 2007; Nour-Eldin and Halkier, 2013)—and is coordinated through a complex network of pathways, including transmembrane transporter–mediated efflux and influx, symplasmic trafficking via plasmodesmata, and vesicle-mediated endocytosis and exocytosis (Verma et al., 2012; Shitan et al., 2014a). Membrane transporters export SMs from source cells into the apoplast or import them into target cells for storage or utilization (Yazaki et al., 2008). These transporters sense concentration changes and regulate dynamic SM levels for accumulation, partitioning, or activation. Manipulating transporter activity has therefore emerged as a powerful strategy for enhancing the accumulation of beneficial metabolites or reducing harmful ones (Zhao, 2015; Liu et al., 2023). Researchers are also developing multifunctional crops by engineering long-distance SM transport systems (Nambiar et al., 2021; Mann et al., 2023). Despite this progress, many aspects remain unresolved, including transporter localization, substrate specificity, affinity, regulation, and coordination of SM transporters in source and sink physiology, particularly in long-distance organ-to-organ transport.

Several studies have identified transporters involved in the spatial distribution of defense and anti-nutritional SMs (Table 1), providing valuable guidance for transporter engineering to enhance desirable SMs or limit the accumulation of undesirable compounds in edible tissues. In this review, we summarize the properties of reported transporters for plant defense and anti-nutritional SMs, focusing on source–sink transporters as engineering targets for crop improvement. Specifically, we address the following questions: (1) How are defense and anti-nutritional compounds transported in plants? (2) Which transporters are involved in SM transport? (3) How can SM transporters be harnessed for crop improvement? (4) What technologies hold promise for characterizing long-distance SM transporters and breeding multifunctional crops? Finally, we discuss challenges that arise in manipulating transporters for crop improvement and potential solutions.

**Table 1:** Transporters involved in the transport of defense and anti-nutritional secondary metabolites in plants.

Family	Gene name	Accession no.	Transport substrate	Tissue expression	Subcellular localization	Transport pathway	Species	Reference
ABC	*CjABCB1 (CjMDR1)*	AB043999	berberine	rhizome xylem	PM	uptake in rhizomes	*C. japonica*	Yazaki et al. (2001); Shitan et al. (2003), (2005)
ABC	*CjABCB2*	AB674325	berberine	cells around rhizome xylem	PM	unloading from xylem	*C. japonica*	Shitan et al. (2013)
ABC	*CjABCB3*	AB674326	berberine	–	–	–	*C. japonica*	Shitan et al. (2013)
ABC	*CrTPT2*	KC511771	catharanthine	leaf epidermis	PM	from epidermis to leaf surface	*C. roseus*	Yu and De Luca (2013)
ABC	*LaABCB11*	–	lycorine	phloem of leaf, bulb, and root	PM	from leaves and bulbs to roots	*L. aurea*	Wang et al. (2021)
ABC	*AmABCB1*	UFQ90028	sanguinarine, berberine	mature seed, pericarp	PM	–	*A. mexicana*	Loza-Muller et al. (2021)
MATE	*NtMATE1*	AB286961	nicotine, hyoscyamine, scopolamine	root	tonoplast	from cytosol to vacuole	*N. tabacum*	Shoji et al. (2009)
MATE	*NtMATE2*	AB286962	nicotine	root	tonoplast	from cytosol to vacuole	*N. tabacum*	Shoji et al. (2009)
MATE	*NtJAT1*	AM991692	nicotine, berberine	leaf, stem, root	tonoplast	sequestration in leaf vacuole	*N. tabacum*	Morita et al. (2009); Yamada et al. (2022)
MATE	*NtJAT2*	AB922128	nicotine, berberine	leaf	tonoplast	sequestration in leaf vacuole	*N. tabacum*	Shitan et al. (2014b)
MATE	*CjMATE1*	LC199487	berberine	rhizome	tonoplast	stored in vacuole	*C. japonica*	Takanashi et al. (2017)
MATE	*CsMATE1*	Csa1G044870	cucurbitacin C	leaf, stem, fruit	tonoplast	from cytosol to vacuole	*C. sativus*	Ma et al. (2023)
MATE	–	Manes.16G007900/Manes.16G008000	cyanogenic glucosides	root and shoot apical meristems	–	–	*M. esculenta*	Ogbonna et al. (2021)
NPF	*CrNPF2.9*	KX372303	strictosidine	leaf epidermis	tonoplast	from vacuole into cytosol	*C. roseus*	Payne et al. (2017)
NPF	*CrNPF2.4/CrNPF2.5/CrNPF2.6*	ALE20039/ALE20040/ALE20041	iridoid glucosides	stem, leaf	PM	into epidermal cells	*C. roseus*	Larsen et al. (2017)
NPF	*GORKY*	Solyc03g120570	α-tomatine	fruit	tonoplast	from vacuole to cytosol	*S. lycopersicum*	Kazachkova et al. (2021)
NPF	*MeCGTR1*	Me15g18400	linamarin	–	–	–	*M. esculenta*	Jørgensen et al. (2017)
NPF	*GTR1*	AT3G47960	glucosinolates	vasculature, mesophyll cells, root cortex	PM	seed loading, movement between cells	*Arabidopsis thaliana*	Nour-Eldin et al. (2012)
NPF	*GTR2*	AT5G62680	glucosinolates	veins	PM	seed loading, import into phloem	*A. thaliana*	Nour-Eldin et al. (2012)
NPF	*GTR3*	AT1G18880	indole glucosinolates	root phloem	–	retained in roots	*A. thaliana*	Jørgensen et al. (2017)
NPF	*BjuGTR1/BjuGTR2*	–	glucosinolates	seed, pod wall	–	from siliques to seeds	*B. juncea*	Nour-Eldin et al. (2017); Nambiar et al. (2021); Mann et al. (2023)
NPF	*BnaC02.GTR2*	BnaC02g42260D	glucosinolates	–	–	from siliques to seeds	*B. napus*	Tan et al. (2022)
NPF	*BnaA06.GTR2*	–	glucosinolates	silique wall	–	from siliques to seeds	*B. napus*	He et al. (2022)
PUP	*NtNUP1*	GU174267	nicotine, vitamin B6	leaf, root (especially root tip)	PM	from apoplast to cytosol	*N. tabacum*	Hildreth et al. (2011); Kato et al. (2015)
PUP	*PUP1*	–	tropane	–	tonoplast	–	*A. belladonna*	Srinivasan and Smolke (2021)
PUP	*CsPUP10.1*	–	caffeine	–	PM	–	*C. sinensis*	Zhang et al. (2022)
PUP	*BUP1*	MH838003	benzylisoquinoline	latex	PM	–	*Papaver somniferum*	Dastmalchi et al. (2019)
UMAMIT	*UMAMIT29*	AT4G01430	glucosinolates	funiculi vasculature, biosynthetic cells, chalazal seed coat	PM	from siliques to seeds	*A. thaliana*	Xu et al. (2023); Sanden et al. (2024)
UMAMIT	*UMAMIT30/31*	AT4G01440/AT4G01450	glucosinolates	funiculi	PM	from siliques to seeds	*A. thaliana*	Xu et al. (2023); Sanden et al. (2024)

PM, plasma membrane.

## Transport of defense and anti-nutritional secondary metabolites in plants

**Figure 1:**
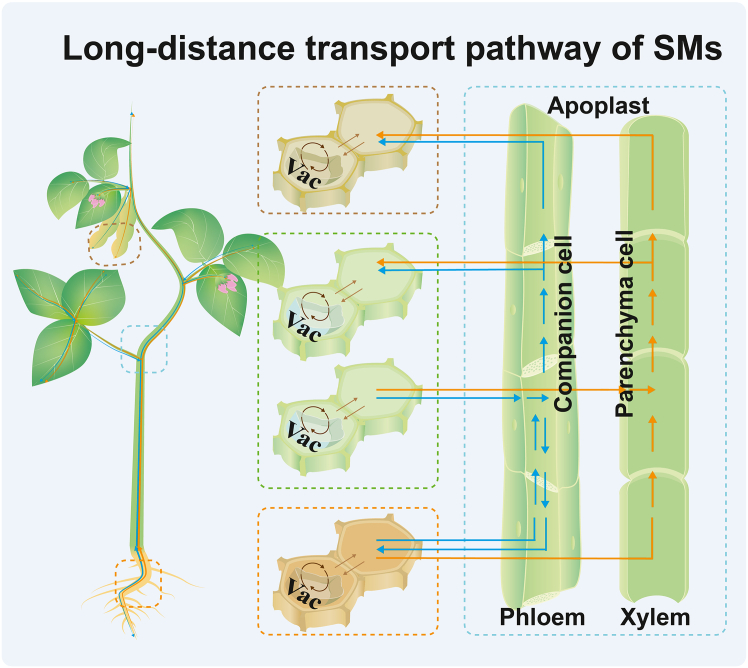
Schematic representation of organ-to-organ long-distance transport pathways of secondary metabolites in plants. Long-distance transport occurs via the phloem (blue arrows) and xylem (orange arrows) pathways, enabling movement of metabolites between organs such as roots, leaves, pods, and seeds. Within individual organs, metabolites move through intracellular routes (e.g., transport between the cytosol and vacuole; dark brown arrows) and intercellular routes (light brown arrows), facilitating short-distance trafficking between biosynthetic and storage sites. Notably, the plasticity of roots as both source and sink underscores the dynamic nature of secondary metabolite (SM) allocation and the potential for bidirectional translocation through the vascular system. Vac, vacuole.

The partitioning of SM synthesis and storage into distinct organelles, cells, and organs is widespread in plants. Lipophilic precursors or intermediates of SMs are typically transported at the intracellular or intercellular level (here referred to as short-distance transport) to balance defensive functions while minimizing autotoxicity. In contrast, hydrophilic SMs such as alkaloids, GSLs, cyanogenic glucosides, iridoid glycosides, and other glycosides are transported across organs via the vascular system (here referred to as long-distance transport). Notably, long-distance transport between source and sink organs through the vascular system is closely integrated with intracellular and intercellular transport processes within those organs (Figure 1). Understanding both the short- and long-distance transport of defensive and anti-nutritional SMs is essential for elucidating the mechanisms of SM transport and for enabling precise manipulation of transporters in crop improvement.

### Short-distance intracellular and intercellular transport of defense and anti-nutritional secondary metabolites

The relocation of metabolites among cellular compartments adds a spatial dimension to plant metabolism and provides an effective mechanism to mitigate self-toxic substances (Shitan, 2016). Intracellular and intercellular transport occur within individual cells or between neighboring cells, regulating the local accumulation, sequestration, or metabolic flux of SMs. For example, cucurbitacin C (CuC), a bitter and defensive triterpenoid, is synthesized and transported within leaf mesophyll cells in cucumber (*Cucumis sativus*) (Shang et al., 2014; Ma et al., 2023). Steroidal glycoalkaloids (SGAs) are transported intracellularly to control toxicity levels in tomato (*Solanum lycopersicum*) fruit (Kazachkova et al., 2021). The predominant and bitter SGA α-tomatine is sequestered into the vacuoles of immature green fruits and later relocated to the cytosol, where it is metabolized into the non-bitter compound esculeoside A during ripening (Kazachkova et al., 2021). This conversion reduces bitterness and toxicity, rendering the fruit safe for consumption. In the medicinal plant *Catharanthus roseus*, the monoterpene indole alkaloid (MIA) pathway is localized across multiple organelles and at least four cell types (St-Pierre et al., 1999; Mahroug et al., 2007). MIA precursorsare synthesized in the cytoplasm and transported into the vacuoles of the leaf epidermis, where biosynthesis of the central intermediate strictosidine and its efflux to the cytosol occur (Payne et al., 2017). This organellar separation likely prevents the accumulation of harmful intermediates. Another well-characterized MIA pathway involves the efflux of catharanthine (an intermediate derived from strictosidine aglycone) from the leaf epidermis to the cuticle (Roepke et al., 2010). Intracellular and intercellular transport pathways involve membrane-transporter-mediated vacuolar sequestration and release, export from the cytosol to the apoplast, vesicle-mediated membrane transport, and symplastic or apoplastic movement between adjacent cells (Halkier and Xu, 2022).

### Long-distance source–sink transport of defense and anti-nutritional secondary metabolites

Long-distance transport of SMs involves the translocation of defensive and anti-nutritional metabolites from source organs (e.g., leaves, stems, pods, and roots) to sink organs (e.g., rhizomes, flowers, fruits, and seeds). Efflux from source to sink organs occurs via two main routes: the xylem and the phloem (Figure 1) (Nour-Eldin and Halkier, 2013). The xylem primarily mediates upward transport from roots to shoots, whereas the phloem distributes metabolites bidirectionally between source and sink organs. For instance, berberine, an antibacterial alkaloid, is synthesized mainly in the lateral roots of *Coptis japonica* and transported through the xylem to the rhizome (Ikezawa et al., 2003; Shitan et al., 2003; 2013). Similarly, nicotine, a well-known alkaloid, is synthesized in the roots of tobacco (*Nicotiana tabacum*) and translocated upward through the xylem to aerial tissues, where it accumulates to deter herbivores (Baldwin, 1989, 2001). In *Arabidopsis thaliana* and other Brassicaceae species, GSLs are synthesized primarily in leaves and pods and transported to seeds via long-distance transport in the phloem (Andersen et al., 2013; Xu et al., 2023). In *Arabidopsis*, aliphatic GSLs move bidirectionally between rosettes and roots through the phloem and xylem (Andersen et al., 2013). Rosettes serve as the main source and reservoir for short-chain aliphatic GSLs, while long-chain aliphatic GSLs are synthesized in both rosettes and roots, with roots acting as the major storage site (Andersen et al., 2013). This spatial division of synthesis and storage indicates tightly regulated bidirectional transport to balance systemic distribution. In cassava (*Manihot esculenta*), cyanogenic glucosides are synthesized in leaves and possibly stems and transported via the phloem to the roots, where they serve as a nitrogen source for amino acid synthesis or are stored in vacuoles to prevent autotoxicity (Jørgensen et al., 2005; Gleadow and Møller, 2014). In narrow-leafed lupin (*Lupinus angustifolius* L.), most quinolizidine alkaloids in seeds are translocated from vegetative tissues (leaves, stems, and pods) to seeds through long-distance phloem transport (Lee et al., 2007; Otterbach et al., 2019).

Long-distance source–sink transport involves specialized vascular loading and unloading mechanisms mediated by plasma membrane (PM)-localized transporters, either importers or exporters, in xylem-adjacent parenchyma cells or phloem companion cells (Nour-Eldin and Halkier, 2013). For SMs to enter the vascular stream, they must first be mobilized from their biosynthetic site, usually in the cytosol, and cross membrane barriers to reach the vascular loading cells. In xylem-mediated transport, SMs enter the apoplast near the xylem and are exported into xylem vessels via transporters located on the PM of xylem parenchyma cells, particularly those mediating efflux into the xylem apoplast (Shitan et al., 2003). In phloem-mediated transport, SMs are imported into companion cells across the PM, often via proton-coupled transporters (Nour-Eldin et al., 2012). Once in the phloem, metabolites move through a regulated symplasmic conduit and may undergo retrieval or recycling en route to sink organs (Chen et al., 2001). Upon arrival at sink organs, SMs are unloaded from the vasculature and further transported across membranes or between cells to reach their destination, such as storage vacuoles, epidermal cells, or other defense-related target sites (Sanden et al., 2024).

## Transporters of defense and anti-nutritional secondary metabolites in plants

**Figure 2:**
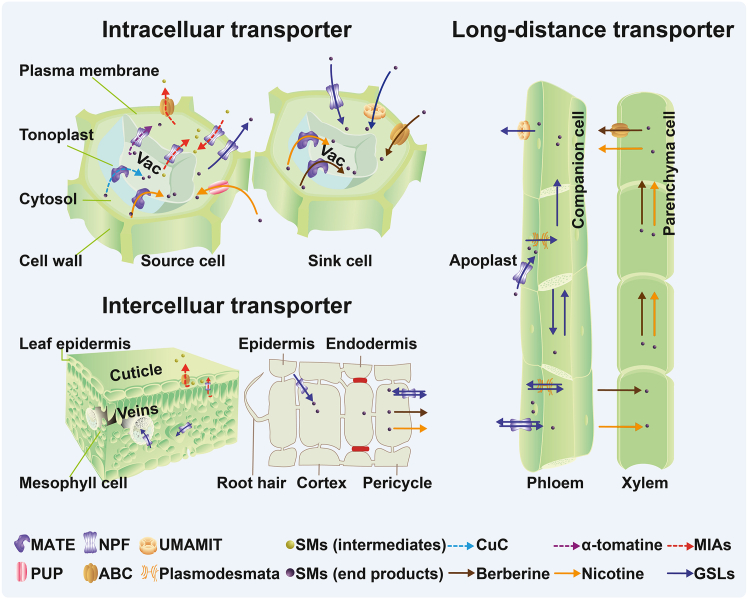
Schematic representation of identified transporters of plant defense and anti-nutritional secondary metabolites. In source cells, NPF and MATE transporters mediate the movement of secondary metabolites (SMs) between the vacuole and cytosol, while ABC, NPF, and PUP transporters facilitate plasma membrane (PM) efflux and uptake at the cellular interface. In sink cells, MATE transporters import SMs into vacuoles for storage, and NPF, UMAMIT, and ABC family members transport SMs across the PM into the cytosol. Intercellular transporters, including NPF and ABC proteins, mediate SM movement between neighboring cells in leaves and roots. Roots act as the source for berberine and nicotine and as one of the sink organs for glucosinolates. Long-distance transport of these three SMs is mediated by xylem and phloem loading and unloading with the help of NPF, UMAMIT, and ABC transporters. Arrows indicate transport dynamics. Vac, vacuole; CuC, cucurbitacin C; MIAs, monoterpene indole alkaloids; GSLs, glucosinolates.

Various mechanisms contribute to the transport and distribution of plant metabolites, including simple diffusion, symplasmic transport through plasmodesmata, vesicle-mediated transport, and substrate-specific membrane transport (Nogia and Pati, 2021). Among these, membrane transporters have emerged as key regulatory nodes and major research targets because of their central roles as gatekeepers of metabolite flux and their potential for genetic and biotechnological manipulation (Lv et al., 2016). These SM transporters are primarily localized to the tonoplast or PM of source and sink cells, as well as to vascular tissues (Figure 2). The main transporter protein families reported include the ATP-binding cassette (ABC) family (Yazaki et al., 2001; Wang et al., 2021), the multidrug and toxic compound extrusion (MATE) family (Shoji et al., 2009; Ma et al., 2023), the nitrate/peptide transporter family (NPF) (Nour-Eldin et al., 2012; Payne et al., 2017), the purine uptake permease (PUP) family (Hildreth et al., 2011; Dastmalchi et al., 2019), and the recently identified “usually multiple amino acids move in and out transporters” (UMAMIT) (Xu et al., 2023; Sanden et al., 2024) (Table 1). To better illustrate their physiological roles, these transporters can be grouped into intracellular or intercellular transporters (e.g., vacuolar-localized) and source–sink long-distance transporters (e.g., vasculature-localized).

### Intracellular and intercellular transporters

Intracellular transporters mediate the import and export of SMs across compartmental boundaries, which is essential for homeostasis, detoxification, and compartmentalized biosynthesis. Tonoplast-localized transporters sequester toxic or storage-bound SMs into vacuoles, while others facilitate remobilization back into the cytosol when metabolites are needed for downstream modification or export. Several members of the MATE and NPF families are located on the tonoplast and act as SM importers or exporters of vacuoles (Figure 2). For example, in cucumber, CsMATE1 is involved in the import of CuC into vacuoles, with its expression co-regulated with CuC biosynthetic genes and transcription factors that directly activate its promoter (Ma et al., 2023). The coordinated biosynthesis and intracellular transport of bitter cucurbitacins may represent a mechanism to regulate the production and spatial partitioning of defensive metabolites while minimizing self-toxicity. In tomato, GORKY from the NPF family is essential for transporting bitter α-tomatine from vacuoles to the cytosol for conversion into the non-bitter esculeoside A (Kazachkova et al., 2021). In *C. roseus*, CrNPF2.9 functions as a vacuolar exporter of strictosidine into the cytosol for downstream modification (Payne et al., 2017).

Transporters at the PM regulate short-distance intercellular movement, either by exporting metabolites to the apoplast or importing them back into the symplasm for further conversion along a biosynthetic pathway, or by transferring them to adjacent storage cells (Halkier and Xu, 2022). This process involves transporters from the ABC and NPF families. For example, the PM-localized ABCG transporter CrTPT2, whose mRNA is predominantly detected in the leaf epidermis, functions as a catharanthine exporter from the epidermis to the leaf surface (Yu and De Luca, 2013). Three other PM-localized CrNPF2.4/CrNPF2.5/CrNPF2.6 transporters of iridoid glucosides, whose genes are expressed in the stem and leaf, are essential for transporting iridoid glucosides (precursors of MIAs) into epidermal cells (Larsen et al., 2017).

### Source–sink long-distance transporters

Source–sink long-distance transporters function at the interface of vascular tissues, enabling the mobilization of metabolites from source to sink organs via the xylem and/or phloem. These transporters contribute to SM allocation by regulating cellular uptake or efflux across membranes during long-distance translocation rather than directly partitioning or distributing SMs over long distances (Atkins and Smith 2007; Andersen et al., 2013). They are often localized at the PM of specialized cell types, such as companion cells in the phloem or parenchyma cells adjacent to xylem vessels. They enable the loading and unloading of SMs into transport streams, often using energy-dependent mechanisms such as proton-coupled symport (NPF family) or ATP-driven export (ABC family) (Shitan et al., 2003, 2013; Nour-Eldin et al., 2012) (Figure 2).

Transporters involved in the long-distance transport of GSLs have been well characterized in plants. In *Arabidopsis*, GSL transporters (GTRs) in the NPF and UMAMIT families play key roles in long-distance GSL transport. These transporters are essential for various processes, including GSL redistribution within leaves, exudation from roots, seed loading, and directed movement to stem S-cells for storage (Nour-Eldin et al., 2012; Madsen et al., 2014; Xu et al., 2017; 2019). They facilitate GSL trafficking among roots, shoots, leaves, pods, and seeds (Hunziker et al., 2019, 2021). In leaves, GTR1 and GTR2 function as PM-localized transporters, primarily expressed in veins, while *GTR1* is also expressed in adjacent mesophyll cells (Nour-Eldin et al., 2012). GSLs are transported across the PM into phloem companion cells in leaves via GTR1 and GTR2, with GTR1 also mediating import into mesophyll cells. In roots, GTR1 and GTR2 are predominantly localized to cortex and vascular cells, contributing to the distribution of indole GSLs between root and shoot (Jørgensen et al., 2017; Xu et al., 2017). *GTR3* is strongly expressed in the companion cells of the root phloem, where it facilitates the retention of indole GSLs in roots (Jørgensen et al., 2017). Additionally, three PM-localized UMAMITs (UMAMIT29, UMAMIT30, and UMAMIT31) are involved in GSL movement from siliques to seeds via funiculus export (Xu et al., 2023). UMAMIT29 is localized in cortex cells and cells adjacent to xylem vessels in the funiculus, as well as in the outer integument and chalazal seed coat. UMAMITs also export GSLs from biosynthetic cells to the apoplast surrounding the vasculature (Sanden et al., 2024).

### Intracellular and intercellular transporters involved in long-distance translocation

Some transporters indirectly support long-distance SM movements, acting through intracellular and intercellular transport rather than vascular pathways. For example, the tonoplast-localized *CjMATE1*—predominantly expressed in rhizomes but also in leaves, petioles, and roots—encodes a protein that facilitates berberine accumulation in vacuoles (Takanashi et al., 2017). Similarly, nicotine transporters NtMATE1 and NtMATE2 are abundant in root tissues, encoding tonoplast proteins that mediate nicotine transport from the cytosol to vacuoles (Shoji et al., 2009). The PM-localized NtNUP1 assists in the movement of apoplastic nicotine into the cytoplasm of root cells (Hildreth et al., 2011). Additionally, tonoplast-localized NtJAT1 and NtJAT2 are responsible for nicotine sequestration in leaf vacuoles (Morita et al., 2009; Shitan et al., 2014b). Although *NtJAT1* is detected in leaves, stems, and roots, *NtJAT2* is expressed specifically in leaves. The expression patterns and localization of these two transporters do not support a role in unloading nicotine from the xylem into leaf cells. Certain membrane transporters, such as GTR1, participate in both long-distance translocation via the vascular system and intercellular transport near the xylem or phloem, reflecting the interconnection between local metabolism and systemic metabolite distribution (Madsen et al., 2014).

## Manipulating transporters for crop improvement

The structural and functional diversity of SMs means that modifying their transport can affect multiple processes in plants. Manipulating SM transporters could regulate metabolite production, direct their accumulation to specific tissues or organs, and enhance resistance to biotic and abiotic stresses, while reducing anti-nutritional compounds in edible parts (Nogia and Pati, 2021). In the following sections, we explore the potential of transporter engineering to influence intracellular and intercellular transport (e.g., catharanthine, glycoalkaloids, and cucurbitacins) and long-distance translocation (e.g., berberine, nicotine, and GSLs) of SMs in plants (Table 2).

**Table 2:** Engineered transporters involved in intracellular (blue), intercellular (orange) and long-distance organ-to-organ (green) transport of defensive and bitter secondary metabolites for crop improvement.

CuC, cucurbitacin C; MIAs, monoterpene indole alkaloids; GSLs, glucosinolates.

### Engineering of defense and bitter secondary metabolite transporters involved in intracellular or intercellular processes

Engineering transporters involved in intracellular and intercellular transport has emerged as a useful strategy for crop improvement by modulating the accumulation of bioactive compounds in plants. These transporters often affect the accumulation of SMs in specific organelles or cells. By controlling the expression of these transporters, plants may alter the yield of end products by regulating metabolic flux toward intermediates or final products. Several studies illustrate this potential. In cucumber, *CsMATE1* knockout mutants showed a significant decrease in the accumulation of bitter CuC in cotyledons (Ma et al., 2023). In tomato, *GORKY* overexpression decreased bitter α-tomatine and increased non-bitter esculeoside A in leaves and fruits (Kazachkova et al., 2021). The success of reducing bitterness to improve crop traits depends on a detailed understanding of transporter function and SM flux at the organelle level. Multiple genetic approaches support roles for these transporters as metabolite valves on the tonoplast. In cucumber, *CsMATE1* knockout reduced vacuolar CuC content by 65%–70% and suppressed the expression of CuC biosynthesis genes, confirming its role as a vacuolar importer and revealing feedback between transport and biosynthesis (Ma et al., 2023). Silencing *GORKY* elevated α-tomatine and reduced esculeoside A in infected tomato fruit, while knockout mutants accumulated upstream SGA intermediates and depleted the final non-bitter product (Kazachkova et al., 2021).

This strategy also applies to defense or pharmaceutically relevant alkaloids such as MIAs. In *C. roseus, CrTPT2* knockdown reduced catharanthine levels on the leaf surface but increased it within leaves (Yu and De Luca, 2013). Conversely, *CrTPT2* overexpression resulted in a fivefold increase in catharanthine in hairy roots (Wang et al., 2019). Silencing *CrNPF2.9* caused a strong increase in strictosidine together with a decrease in MIA end products, consistent with the role of CrNPF2.9 as a vacuolar exporter (Payne et al., 2017). Overexpression could potentially be used to increase MIA accumulation in *C. roseus*.

These examples collectively highlight that manipulating intracellular and intercellular transporters can reduce the accumulation of toxic intermediates or undesirable bitter compounds while increasing yields of pharmacologically important SMs. To minimize potential risks associated with transporter engineering, such as pleiotropic developmental defects observed in *GORKY*-overexpressing tomato (e.g., dwarfism and floral deformities) and *CrNPF2*.9-silenced *C. roseus* (leaf cell death), tissue-specific promoters or backcrossing into elite lines could be employed. Furthermore, the physiological roles of some SMs pose trade-offs that must be considered in crop breeding. For instance, CuC imparts bitterness to edible tissues but also contributes to plant defense. However, it remains unclear whether all CuC in cucumber fruits is synthesized locally or partly transported from leaves, and CuC levels in *CsMATE1* mutant fruits have yet to be analyzed (Shang et al., 2014; Ma et al., 2023).

### Manipulation of transporters that mediate long-distance organ-to-organ transport

Using transporters that mediate long-distance organ-to-organ transport to regulate the distribution of defense and bitter SMs remains relatively underexplored. In tobacco, silencing of *NtMATE1/2* through RNA interference (RNAi) failed to alter nicotine profiles in roots and leaves (Shoji et al., 2009). Downregulation of *NtNUP1* by RNAi decreased nicotine accumulation in roots and leaves without affecting root-to-shoot translocation (Hildreth et al., 2011). These findings suggest that although certain transporters mediate the movement of SMs that are ultimately distributed via long-distance pathways, their direct roles may be restricted to cellular uptake, intracellular sequestration, or local export within source or sink tissues rather than vascular loading or unloading.

Further functional characterization revealed that nicotine transporters are not strictly nicotine specific but exhibit broad substrate specificity. For example, several of these transporters efficiently transported hyoscyamine, NtJAT1 and NtJAT2 transported berberine, and NtNUP1 also transported vitamin B6 (Kato et al., 2015). Such substrate promiscuity poses a challenge for targeted engineering and suggests a need for context-dependent validation of transporter functions. Although heterologous expression of *NtJAT1* in *Escherichia coli* increased alkaloid production by 14-fold, its *in planta* function remains unclear, raising questions about interaction with endogenous transport systems and underscoring the complexity of engineering SM transporters (Yamada et al., 2022). The limited success in manipulating such transporters further underscores the need to identify and characterize long-distance transporters that could influence source–sink efflux. For example, manipulation of PM-localized CjABCB1 or CjABCB2 may affect root-to-rhizome berberine translocation in *C. japonica*, as both are highly expressed in rhizome xylem and have been implicated in berberine uptake (Shitan et al., 2003; 2013). However, in transgenic plants with co-suppressed *CjABCB1*, berberine levels were unexpectedly reduced in leaves and petioles (non-primary accumulation sites) as well as in roots (source organ) (Shitan et al., 2005). Due to the lack of expression and metabolite data from the rhizome (sink organ), it remains unclear whether xylem unloading is altered in this tissue.

### Inhibition of long-distance transport produces “low-glucosinolate” seeds with defense potential

Following advances in engineering short-distance SM transporters, the potential for harnessing long-distance transporters is becoming increasingly evident. Also, the historical reduction of bitterness in plants may have overlooked the value of these compounds (Liu et al., 2022). Emerging research underscores that bitter SMs such as GSLs and alkaloids not only defend plants against pests and pathogens but also possess therapeutic potential for humans, including anti-inflammatory, antioxidant, and antitumor activities (Drewnowski and Gomez-Carneros, 2000; Liu et al., 2022). Given their dual role as plant protectors and nutraceuticals, engineering SM long-distance transporters should focus on retaining their benefits in vegetative tissues while minimizing their presence in edible organs.

By targeting GSL long-distance transporters such as GTR1, GTR2, GTR3, or UMAMIT29/30/31, researchers can block GSL loading into seeds while maintaining biosynthesis and storage in leaves (Kumar et al., 2017; Liu et al., 2020). In *Arabidopsis*, knockout mutants of *GTR1* and *GTR2* show distinct roles in GSL allocation: *gtr1* mutants retain wild-type seed GSL levels, whereas *gtr2* mutants display a significant reduction (48% ± 11%) (Nour-Eldin et al., 2012). The *gtr1 gtr2* double mutant eliminates detectable seed GSLs, highlighting the dominant role of GTR2 in seed loading and a compensatory role for GTR1. This may be linked to GTR1’s additional role in mesophyll cells. In *gtr3* mutants, indole GSLs are significantly lower in roots but elevated in the rosette. Furthermore, the *gtr1 gtr2* double mutant showed increased rosette indole GSLs, which was fourfold that of the *GTR3* knockout mutant (Jørgensen et al., 2017). Beyond the GTR family, *UMAMIT29/30/31* transporters are highly expressed in funiculi—the only vascular connections between the silique septum and the seed (Xu et al., 2023; Sanden et al., 2024). Mutant analysis shows comparable silique GSLs but an 80% reduction in seed GSLs in *umamit29* and less than 5% of seed GSLs in the *umamit29 umamit30 umamit31* triple mutant compared with the wild type, demonstrating nonredundant functions in seed loading (Xu et al., 2023). The divergent outcomes of manipulating these transporters, from tissue-specific redistribution (e.g., GTR3) to near-complete elimination of seed GSLs (UMAMIT29/30/31), reflect distinct biochemical roles, spatial expression, and compensation. Although manipulating these transporters alters GSL efflux from source to sink organs, the impact of increased GSLs in the source on plant defense has not been specifically addressed.

In *Brassica juncea* and *Brassica napus*, research on GTR manipulation has focused on multiple *GTR1* and *GTR2* homologs. In *B. juncea*, four functional *BjuGTR2* homologs were analyzed by TILLING (Nour-Eldin et al., 2017). Quadruple mutations in *BjuGTR2* led to a 62% ± 10% reduction in seed GSL content compared to the wild type, with a GSL concentration of 44 ± 11 μM/g dry weight (DW), exceeding the canola standard of 30 μM/g DW (Nour-Eldin et al., 2017). RNAi-mediated knockdown of *BjuGTR2* resulted in a >50% reduction in seed GSL levels (30.06 μM/g DW) while simultaneously increasing GSL concentrations in the leaves and pods compared with wild-type plants, enhancing resistance against generalist pests (Nambiar et al., 2021). However, *BjuGTR1*-silenced and *BjuGTR1/GTR2-*co-silenced lines showed reduced GSL levels in seeds, leaves, and pods, compromising defense against herbivores (Nambiar et al., 2021). In *B. napus*, Tan et al. (2022) mutated *GTR2* homologs using CRISPR–Cas9-based editing. Association mapping identified *BnaC02.GTR2* as the key homolog regulating seed GSL transport. Editing *BnaC02.GTR2* together with three other *GTR2* homologs reduced seed GSLs by 86.85% to 18.21 μM/g (Tan et al., 2022). These edits also reduced leaf GSL content, 1000-seed weight, and seed size, and altered seed amino acid, sugar, and fatty acid composition (Tan et al., 2022). In contrast, a loss-of-function mutation in *BnaA06.GTR2* reduced seed GSLs by 76.05% to approximately 30 μM/g (He et al., 2022). Notably, these mutants exhibited no apparent changes in morphology or 1000-seed weight but lower-than-normal GSL levels in developing silique walls (He et al., 2022). Recently, simultaneous mutation of multiple *BjuGTR1* and *BjuGTR2* homologs in *B. juncea* using CRISPR–Cas9 reduced seed GSL to <30 μM/g DW in T2 and T3 plants, with high leaf GSL content (>70 μM/g DW) and relatively high pod GSLs (30–70 μM/g DW) (Mann et al., 2023). Despite these changes, the mutants maintained defense response and seed quality, underscoring the promise of modifying SM long-distance transporters for crop improvement.

Research on GSL transport in *Arabidopsis* and *Brassica* provides critical insight into how SMs, such as GSLs, are mobilized and highlights the importance of understanding transport mechanisms for breeding. By manipulating long-distance transporters responsible for vascular loading and unloading and relocation in source and sink organs, it may be possible to improve seed quality while maintaining effective defense. Key factors for success include identifying specific transporters and selecting appropriate editing strategies. For example, GTR1 functions as a compensatory and intercellular transporter (Nour-Eldin et al., 2012). Thus, editing *GTR2* and *BjuGTR2* rather than *GTR1* and *BjuGTR1* has been more effective for reducing seed GSLs while preserving defense in source tissues (Nambiar et al., 2021). Additional considerations contribute to the successful manipulation of SM transport. These include source–sink relationships, transport directionality, subcellular localization, specific cell types in which transporters act, and whether they serve as gatekeepers of long-distance vascular transport (e.g., phloem or xylem loading). Substrate specificity also affects selectivity and feasibility; for instance, GTR1 contributes to jasmonate transport in *Arabidopsis*, which is important for stress signaling (Ishimaru et al., 2017). Therefore, engineering strategies targeting GTR1 may cause unintended effects, such as reduced stress tolerance. In species with multiple homologs, such as *Brassica*, decisions regarding which transporters to edit—whether individually or in combination—as well as the choice of gene-editing method and target sites, can profoundly influence the phenotype, sometimes leading to adverse effects. Therefore, breeding strategies should undergo iterative optimization to achieve the desired crop improvements. For example, because *GTR2* has multiple homologs in *Brassicaceae*, selecting a homolog with a suitable expression pattern is important (Mann et al., 2023). He et al. (2022) edited *BnaA06.GTR2*, whose expression mirrors the GSL accumulation pattern, to mitigate the adverse effects on morphology and 1000-seed weight observed in multi-homolog mutations in *B. napus*.

## Technologies and frameworks for discovering and characterizing long-distance secondary metabolite transporters for crop improvement

**Figure 3:**
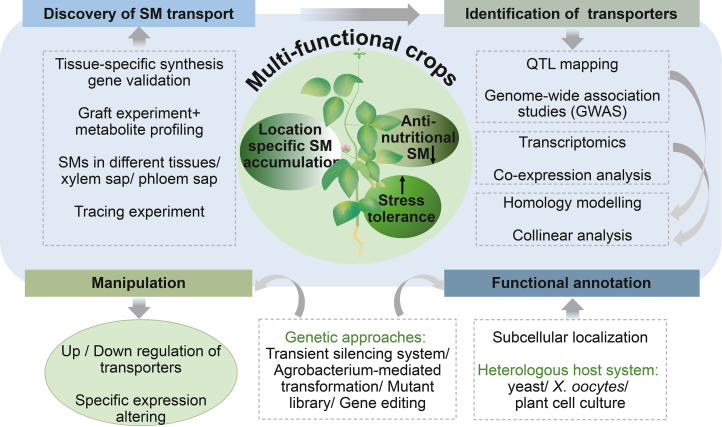
Schematic illustration of approaches for discovering, identifying, and functionally annotating plant secondary-metabolite long distance transporters, and of transporter engineering models and applications for crop improvement.

Manipulating intra- and intercellular transporters involved in both short-distance and long-distance SM transport has clarified transporter functions and underscored the importance of harnessing SM transporters in crop improvement. Compared with intra- and intercellular transporters, long-distance transporters hold greater potential for breeding multifunctional crops that enhance defense while minimizing harmful or anti-nutritional compounds in seeds. It then becomes possible to manipulate transporters to achieve specific crop improvement goals with precision. In the following sections, we explore the technologies and frameworks for the discovery and characterization of long-distance transporters of SMs for crop improvement (Figure 3; Table 3).

**Table 3:** Experimental tools and techniques for investigating and modifying long-distance transport of plant secondary metabolites (SMs).

Purpose	System/method	Tools and techniques	Functional outcome
Discovery of long-distance SM transport	tissue-specific biosynthetic gene validation	RT–qPCR, GUS reporter assay	identification of SM biosynthesis site
	metabolite profiling	LC–MS, HPLC, GC–MS	SM quantification
	grafting experiments	scion/rootstock combinations	determination of transport direction
	xylem/phloem sap analysis	EDTA-facilitated exudation	direct evidence of transport
	tracing experiments	isotopic labeling, fluorescent tracers	real-time transport monitoring
Identification of transporters	QTL mapping/GWAS	SNP arrays, linkage analysis	locus identification
	transcriptomics	RNA-seq, RT–qPCR	tissue- and stage-specific candidate identification
	homology modeling/collinearity	sequence alignment, phylogenetic analysis	identification of orthologs in non-model species
	co-expression analysis	WGCNA, clustering	co-regulated gene prediction
Functional annotation	subcellular localization	organelle marker fusion, confocal microscopy	determination of membrane localization
	cell-/tissue-specific expression analysis	GUS assay, *in situ* hybridization	resolves of tissue- and cell-level distribution
	*X. laevis* oocyte system	microinjection, voltage clamp, uptake assays	transporter screening, substrate specificity
	yeast system	mutant complementation, radiolabeled substrates	substrate validation, heterologous SM biosynthesis
	plant cell culture	suspension cultures, protoplasts	uptake analysis in a plant background, heterologous SM biosynthesis
	transient gene silencing system	VIGS	rapid knockdown
Functional annotation and manipulation	*Agrobacterium*-mediated transformation	overexpression, RNAi, tissue-specific promoters	precisely controlled expression
	mutant library	EMS mutagenesis, TILLING	high-throughput mutant screening
	gene editing	CRISPR–Cas9	precise gene knockout or editing

GUS, β-glucuronidase; LC–MS, liquid chromatography–mass spectrometry; HPLC, high-performance liquid chromatography; GC–MS, gas chromatography–mass spectrometry; EDTA, ethylenediaminetetraacetic acid; WGCNA, weighted gene co-expression network analysis; QTL, quantitative trait locus; GWAS, genome-wide association study; SNP, single-nucleotide polymorphism; RNAi, RNA interference; EMS, ethyl methanesulfonate; TILLING, targeting induced local lesions in genomes; VIGS, virus-induced gene silencing; CRISPR–Cas9, clustered regularly interspaced short palindromic repeats–CRISPR-associated protein 9.

### Discover long-distance transport pathways and related transporters

Building on the successful examples discussed above, the primary question is whether SMs are synthesized *in situ* or transported from other tissues. Several methods can be employed to confirm the existence of long-distance SM transport and clarify source–sink relationships (Table 3). One widely used strategy is to confirm tissue-specific expression patterns of SM biosynthetic genes to identify the primary site of SM biosynthesis (Shoji et al., 2002). Grafting experiments between SM-producing and non-producing genotypes, combined with sap metabolite profiling, can reveal whether SMs move across graft junctions, providing direct evidence of long-distance transport (Xu et al., 2023). Quantitative analysis of SM intermediates or final products across tissues and in xylem or phloem sap offers insight into transport direction and potential vascular loading (Lee et al., 2007). Metabolic tracing experiments (e.g., isotope or stable-label tracking) can monitor real-time movement of SMs through plant tissues (Chokkathukalam et al., 2014). Together, these techniques determine whether a metabolite is produced and retained locally or redistributed via vascular networks. Confirming the existence, directionality, and tissue specificity of such transport is essential for identifying source and sink organs and represents the first step in designing effective transporter-based strategies for crop improvement.

While long-distance SM transport has been documented in species such as lupins and cassias, identifying and functionally validating candidate transporters remains challenging. In particular, integrating advanced molecular and genetic approaches is essential for the identification of these transporters (Figure 3). Recent studies have applied quantitative trait locus mapping and genome-wide association studies, alone or together with RNA sequencing analysis followed by RT–qPCR validation, to predict and identify potential transporters (Tan et al., 2022). For example, two MATE transporters identified through genome-wide association studies showed differential expression between fibrous and storage roots, highlighting their potential role in the transport of cyanogenic glucosides (Ogbonna et al., 2021). Transcriptomic analysis indicated that *LaABCB11, which* encodes a PM-localized lycorine transporter in *Lycoris aurea*, was primarily expressed in the phloem of leaves, bulbs, and roots (Wang et al., 2021). Co-expression analysis with known biosynthetic pathway genes also helps identify co-regulated transporters involved in metabolite transport (Hildreth et al., 2011; Kato et al., 2015; Jørgensen et al., 2017; Yamada et al., 2022). Using these approaches, Srinivasan and Smolke (2021) identified a tropane alkaloid transporter, PUP1, in *Atropa belladonna*, a vacuolar, tonoplast-associated transporter (Srinivasan and Smolke, 2021). Homology modeling and collinearity analyses of known SM transporters offer a straightforward route to nominate candidates in other crops (Ma et al., 2023). These methods identified CsPUP10.1 in *Camellia sinensis*, suggesting that this PM-localized transporter is involved in caffeine intracellular transport (Zhang et al., 2022). Moreover, AmABCB1, an ortholog of CjABCB1 in *Argemone mexicana*, possessed uptake and efflux activities for sanguinarine and berberine in yeast assays (Loza-Muller et al., 2021).

### Inferring transporter function through localization analyses

Once candidate SM transporters are identified, subcellular and tissue-level localization analyses provide critical clues to their roles, especially for distinguishing short-distance from long-distance functions. Confocal microscopy with organelle marker fusions defines the targeted membrane or organelle and thus the transporter’s mode of action (Payne et al., 2017). High-resolution cell- or tissue-specific expression analysis using β-glucuronidase reporters or *in situ* hybridization reveals where and when the transporter gene is expressed, linking it to developmental processes or key transport barriers (Shoji et al., 2009; Sanden et al., 2024). These approaches can be combined to map transporter activity spatially and generate strong preliminary evidence for physiological roles. The PM is a key site for long-distance transporters because it mediates export to or import from the apoplast (Halkier and Xu, 2022). Particular attention should be given to vascular tissues as metabolic efflux pathways and vascular connections between sinks and sources, such as funiculi (Xu et al., 2023). These localization analyses form a foundation for inferring function and selecting candidates for physiological and genetic validation.

### Heterologous expression systems as powerful tools for studying secondary metabolite transporters

Heterologous expression systems such as *Xenopus laevis* oocytes and yeast strains are indispensable for characterizing SM transporter function, including substrate specificity, ion coupling, and transport direction (efflux or uptake). The *X. laevis* oocyte system has been instrumental in identifying the first long-distance GTRs (Nour-Eldin et al., 2012) and in defining substrate specificity under controlled intracellular conditions (Pike et al., 2019). The yeast (*Saccharomyces cerevisiae*) system is widely used because it is easy to manipulate and has low endogenous transport background. When a plant transporter is expressed in yeast, changes in metabolite accumulation can indicate its substrate specificity and the direction of transport (Morita et al., 2009). In addition, plant-based systems such as tobacco BY-2 cells, other suspensions, and protoplasts offer more native-like contexts. For example, GSL uptake assays in cotton cell suspensions (Nambiar et al., 2021) and confocal imaging of transporter activity in mesophyll protoplasts (Hang et al., 2024) have expanded the toolkit. Choice of system should match coupling mechanism. For example, H^+^ -driven MATE antiporters can be assayed in oocytes using voltage clamp (Ma et al., 2023). H^+^ symport PUP transporters are often better studied in yeast due to stable pH gradients (Hildreth et al., 2011; Dastmalchi et al., 2019), whereas the ATP-driven ABC family transporters can be characterized in both yeast and oocytes (Lefèvre and Boutry, 2018). When the focus is on metabolite compartmentation, plant cell systems can provide more informative insights. Beyond functional studies, heterologous expression, especially in plant cell cultures, supports synthetic biology by enabling reconstitution of transport modules to improve metabolite flux, secretion, or storage in microbial or plant chassis (Wu et al., 2021).

### Functional validation of secondary metabolite transporters and genetic manipulation for crop improvement

Experimental validation of SM transporters employs RNAi, overexpression, virus-induced gene silencing, mutant library screening, and CRISPR–Cas9 editing. RNAi and overexpression are typically performed via *Agrobacterium*-mediated transformation, using *A. tumefaciens* for stable transformation and *A. rhizogenes* for roots (Ricigliano et al., 2016). Transformation efficiency remains a limitation for some crops, such as lupin (Uhde-Stone et al., 2005; Sbabou et al., 2010; Cheng et al., 2011). Virus-induced gene silencing offers faster validation (Mancinotti et al., 2021), but its transient nature poses challenges: source–sink phenotypes must be observed before silencing diminishes, and assessing seed- or fruit-related traits can be difficult because silencing duration may not match reproductive cycles. Shortening the reproductive period and extending silencing duration may help address this issue.

Gene silencing can markedly reduce expression (knockdown) but may not fully eliminate it (Alagoz et al., 2016). Mutant libraries combined with TILLING can yield loss-of-function alleles or new variants for functional studies and trait improvement, but the random, unpredictable nature of such mutants requires labor-intensive, time-consuming screening (Sikora et al., 2011). For more a thorough understanding of gene function and targeted modification of specific metabolites, gene-editing tools such as CRISPR–Cas9 are recommended (Zhang et al., 2021). Optimizing CRISPR technology is essential for accommodating the delivery method in recalcitrant and difficult-to-regenerate species such as lupin (Pigeaire et al., 1997). For example, a model allotetraploid tobacco host was engineered to undergo single, multiplex, and chromosomal deletions at a high frequency using a negative-strand RNA virus-based vector for DNA-free delivery of the entire CRISPR–Cas9 cassette (Ma et al., 2020). Additional delivery methods using functionalized nanoparticles have been developed in cotton, sunflower, and lily, enabling transformation without *in vitro* regeneration (Zhao et al., 2017). Functionalized magnetic particles have further enhanced transformation efficiency, accelerating breeding and selection (Watson et al., 2018; Lew et al., 2020). Advances in nanoparticle technologies have also increased CRISPR–Cas9 efficiency, enabling transformation in species previously considered difficult (Ma et al., 2021). These genetic approaches not only validate SM transporters but also allow their manipulation for diverse breeding purposes. In particular, subcellular-level phenotypic analysis is required when whole-organ SM quantification cannot resolve transporter functions, especially for proteins mediating compartmentalized transport (e.g., vacuolar importers or exporters). For example, isolating protoplasts and vacuoles for CuC quantification in *CsMATE1* mutants has provided more precise insight into transporter functionality (Ma et al., 2023), highlighting the need for higher-resolution assays to characterize transporters involved in specific subcellular trafficking pathways.

In species such as narrow-leafed lupin, long-distance transport of defense SMs has been documented (Lee et al., 2007; Otterbach et al., 2019), but the specific transporters involved have yet to be identified. While various candidate transporters for defense SMs are proposed in Table 1, their functions require further validation. Using advanced tools, SM transporters can be identified, validated, and modulated through upregulation, downregulation, or targeted expression to enhance metabolite production, improve tissue- or organ-specific accumulation, boost stress tolerance, reduce anti-nutritional compounds, and facilitate the breeding of multifunctional crops (Figure 3).

## Concluding remarks and future perspectives

Membrane-localized transporters allocate numerous SMs within and between cells, move intermediates, and facilitate long-distance transport between organs. Although only a limited number of SM transporters have been functionally characterized in plants, recent evidence highlights their essential roles in directing metabolites to sites of synthesis and storage and in influencing overall plant metabolism and stress responses. These findings underscore the potential of transporters as targets for genetic engineering to enhance SM production, grain quality, and plant performance under environmental stresses. For transporter engineering to advance, functional validation and the development of effective modification strategies for SM transporters remain major challenges. This review focused on advances in understanding the transport of defense-related SMs, including alkaloids, GSLs, and selected bitter compounds. Systematic characterization of their short-distance and long-distance pathways, including SM exporters and importers on vacuoles, PMs, and vascular tissues, will deepen our understanding of source–sink relationships and metabolite flux. We also examined both successful and unsuccessful transporter manipulations to identify factors that influence outcomes. We emphasize the importance of selecting and targeting key long-distance transporters for the development of multifunctional crops with enhanced nutritional value, reduced toxicity, and maintained stress tolerance. Finally, we propose a workflow for exploring long-distance transport mechanisms of valuable metabolites to facilitate the discovery and functional validation of novel transporters. This knowledge and these strategies can be extended to other economically important plants, such as lupin, cassava, almond, and bitter melon, to reduce anti-nutritional compounds in edible parts while enhancing stress resilience.

Despite recent progress, significant challenges remain. A major limitation of genetic interventions is the unpredictability of outcomes due to incomplete characterization of transporter networks. Many transporters exhibit substrate promiscuity (transport of multiple, unrelated metabolites), functional redundancy (compensation by homologs), or pleiotropic effects (unintended changes in agronomic traits after manipulation). These complexities highlight the need for systematic approaches to resolve transporter specificity, regulation, and interactions. Integrating multi-omics data, for example transporter expression patterns coupled with metabolite flux maps, will be key to deciphering transport networks and regulatory hubs. Emerging platforms are poised to accelerate the discovery and characterization of SM transporters. High-throughput screening of transporters in yeast enables rapid functional testing of large gene sets (Groszmann et al., 2023). Mass spectrometry imaging provides spatial insights into transport routes (Buchberger et al., 2018). Single-cell RNA sequencing reveals cellular expression maps in complex tissues such as the xylem (Tung et al., 2023).

Additionally, AI and machine learning models offer predictive power for identifying transporter structures and substrates and for inferring co-expression patterns in gene networks (Theodoris et al., 2023; Kroll et al., 2024). Together, these platforms will support the construction of comprehensive transporter interaction networks that uncover redundancy, compensatory mechanisms, and source–sink dynamics governing SM distribution. Precision engineering approaches, particularly those based on CRISPR technology, should be further developed to enable targeted modulation of transporter activity across a wider range of species with specific spatial and temporal control, while minimizing off-target effects and improving metabolic control. By overcoming these challenges, engineering of SM transporters can move beyond incremental gains toward crops with tailored metabolite profiles that balance yield, defense, and nutritional quality. This will support modern breeding strategies and offer sustainable solutions to food security and environmental challenges.

## Funding

This work was supported by the Australian Grains Research and Development Corporation (UMU2306-008RSX and UMU2404-007RTX).

## Acknowledgments

The author acknowledges financial support from a Murdoch International Postgraduate Scholarship at Murdoch University.

## Author contributions

C.L. conceptualized the manuscript. C.X. drafted the manuscript and prepared all tables and figures. C.L., G.Z., and T.H. revised the manuscript, tables, and figures. All authors approved the final version of the manuscript for submission.

## Supplemental information

Document S1. Supplemental Table 1

Document S2. Article plus supplemental information
