# Supplementary material for: Transport of secondary metabolites in plants: Mechanistic insights and transporter engineering for crop improvement
Source: Plant Commun. 2025 Sep 25;6(12):101536. doi: 10.1016/j.xplc.2025.101536 (PMC12744758; doi:10.1016/j.xplc.2025.101536)
Supplement: Document S1. Supplemental Table 1 [file mmc1.pdf]

Plant Communications, Volume *6*

## Supplemental information

# Transport of secondary metabolites in plants: Mechanistic insights and transporter engineering for crop improvement

**Chunsheng Xiao, Gaofeng Zhou, Tianhua He, and Chengdao Li**

**Supplementary Table**

TableS1. List of abbreviations and their definitions.

| Abbreviation | Full Term | Description |
|---|---|---|
| ABC | ATP-binding Cassette | Membrane proteins that transport numerous substrates across membranes, including secondary metabolites, via ATP hydrolysis. |
| CuC | Cucurbitacin C | Triterpenoid compound found in cucurbit plants (e.g., cucumber, melon) that has strong antifeedant properties and contributes to plant defense. |
| GLSs | Glucosinolates | Sulfur- and nitrogen-containing secondary metabolites that primarily present in *Brassicaceae* plants and affect crop flavor, nutrition and defense ability |
| GTRs | Glucosinolate transporters | Specific transport proteins belong to NPF family that mediate the long-distance translocation of glucosinolates. |
| MATE | Multidrug and toxic compound extrusion | Transport proteins that mediate the efflux of secondary metabolites using proton gradients. |
| MIAs | Monoterpene indole alkaloids | Secondary metabolites derived from tryptamine and monoterpenes, known for their medicinal properties and commonly found in Catharanthus roseus. |
| NPF | Nitrate peptide family | Transporters family responsible for transporting nitrate and peptides, plant hormones and secondary metabolites. |
| PUP | Purine uptake permeases | Membrane transporters originally identified for purine transport and also mediate uptake of secondary metabolites. |
| SGAs | Steroidal glycoalkaloids | Secondary metabolites containing nitrogen found in Solanaceae species (e.g., tomato and potato). |
| SMs | Secondary metabolites | Organic compounds produced by plants that play critical roles in defense, signaling, and environmental adaptation. |
| UMAMITs | Usually multi-amino acids move in and out transporters | Transport family participate in amino acid exchange between cells and play a role in transporting secondary metabolites as well. |
