# Supplementary material for: Transport of secondary metabolites in plants: Mechanistic insights and transporter engineering for crop improvement
Source: Plant Commun. 2025 Sep 25;6(12):101536. doi: 10.1016/j.xplc.2025.101536 (PMC12744758; doi:10.1016/j.xplc.2025.101536)
Supplement: Document S2. Article plus supplemental information [file mmc2.pdf]

Plant Communications
Review article

CellPress
Partner Journal

Transport of secondary metabolites in plants: Mechanistic insights and transporter engineering for crop improvement

Chunsheng Xiao[1,2], Gaofeng Zhou[1], Tianhua He[1] and Chengdao Li[1,2,3,*]

[1]Western Crop Genetic Alliance, Murdoch University, Perth, WA 6150, Australia

[2]State Agricultural Biotechnology Centre (SABC), College of Science, Health, Engineering and Education, Murdoch University, Perth, WA 6150, Australia

[3]Department of Primary Industry and Regional Development, Government of Western Australia, South Perth, WA 6155, Australia

***Correspondence: Chengdao Li (c.li@murdoch.edu.au)**

https://doi.org/10.1016/j.xplc.2025.101536

ABSTRACT

Secondary metabolites (SMs) are essential for plant survival and adaptation, playing multiple roles in ecological interactions such as defense and stress tolerance. Specialized transporters relocate SMs from their synthesis sites to defense tissues or storage organs. The spatiotemporal distribution of defense-related SMs is a key determinant of plant fitness. However, the accumulation of anti-nutritional SMs in crop seeds or fruits can pose health risks to humans and livestock. Recent advances have revealed the critical role of SM transporters in optimizing metabolite allocation. This review examines the transport mechanisms of both defense and anti-nutritional SMs, with a particular focus on long-distance transporters that regulate source–sink dynamics and their potential applications in agricultural biotechnology. We highlight innovative strategies to manipulate transporter activity, ranging from multi-omics integration to precision engineering, and discuss how these approaches can be used to design crops with enhanced defense capacity, increased levels of beneficial compounds, and improved palatability of seeds and fruits. Finally, we outline emerging technologies and conceptual frameworks for discovering and characterizing long-distance SM transporters for crop improvement. Transporter-focused strategies offer promising solutions to global agricultural challenges and provide new opportunities for advancing crop improvement in the context of global food security.

Key words:: secondary metabolites, transporters, long-distance transport, genetic manipulation, crop improvement

Xiao C., Zhou G., He T., and Li C. (2025). Transport of secondary metabolites in plants: Mechanistic insights and transporter engineering for crop improvement. Plant Comm. **6**, 101536.

INTRODUCTION

Plants produce specialized secondary metabolites (SMs) that enable them to defend against stress and adapt to challenging environments (Isah, 2019). Defense-related SMs, including alkaloids, glucosinolates (GSLs), flavonoids, terpenoids, and phenolic compounds, play vital roles in plant responses to abiotic and biotic stresses (Steppuhn et al., 2004; Freeman and Beattie, 2008; Clay et al., 2009). In addition to their defensive functions, these metabolites exhibit significant bioactive properties and hold considerable pharmaceutical potential. They are widely used in medicines, phytomedicines, nutraceuticals, fragrances, dyes, flavorings, and pesticides (Anokhina et al., 2008; He and Giusti, 2010; Riaz et al., 2023). However, certain SMs in fruits and grains can be bitter or toxic, exhibiting teratogenic or anticholinergic effects that render them unsuitable for human or livestock consumption (Wink, 2010; Wang et al., 2024).

In crop breeding, a major challenge is balancing two objectives: maintaining or enhancing stress tolerance through the accumulation of valuable defense-related SMs while improving the quality of edible plant parts by reducing anti-nutritional SMs (Dwivedi et al., 2021; Qin et al., 2023). A deeper understanding of SM metabolism—particularly the transport pathways that determine their spatiotemporal distribution—is crucial for reconciling these goals. Recent advances in SM transport biology have created new opportunities for the precise engineering of SM allocation,

 Crown Copyright © 2025 Published by Elsevier Inc. on behalf of CAS Center for Excellence in Molecular Plant Sciences, Chinese Academy of Sciences, and Chinese Society for Plant Biology.
This is an open access article under the CC BY license (http://creativecommons.org/licenses/by/4.0/).

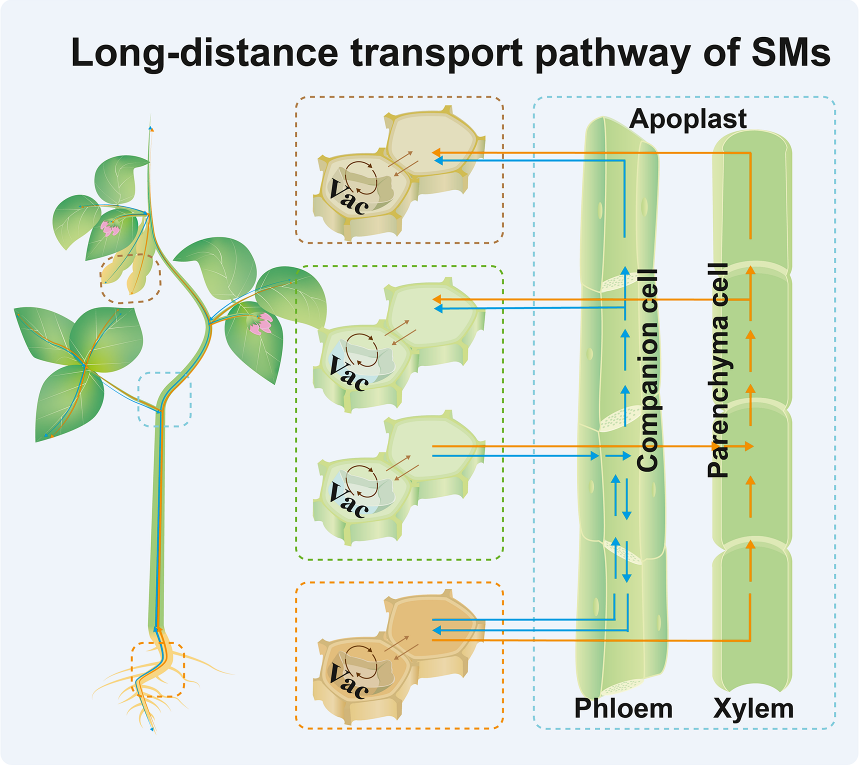

Figure 1. Schematic representation of organ-to-organ long-distance transport pathways of secondary metabolites in plants.
Long-distance transport occurs via the phloem (blue arrows) and xylem (orange arrows) pathways, enabling movement of metabolites between organs such as roots, leaves, pods, and seeds. Within individual organs, metabolites move through intracellular routes (e.g., transport between the cytosol and vacuole; dark brown arrows) and intercellular routes (light brown arrows), facilitating short-distance trafficking between biosynthetic and storage sites. Notably, the plasticity of roots as both source and sink underscores the dynamic nature of secondary metabolite (SM) allocation and the potential for bidirectional translocation through the vascular system. Vac, vacuole.

paving the way for crop improvement (Nogia and Pati, 2021; Bai et al., 2024).

Most defense-related and anti-nutritional SMs are synthesized in source organs (e.g., leaves, stems, pods, and roots) and subsequently transported to sink organs (e.g., rhizomes, flowers, fruits, and seeds), where they perform protective and other biological functions (Nour-Eldin and Halkier, 2009; Verma et al., 2012; Jørgensen et al., 2015; Otterbach et al., 2019). This spatial separation requires tightly regulated transport mechanisms to ensure the precise allocation of SMs to target cells and tissues. Transport occurs at multiple levels—including intracellular, intercellular, and long-distance organ-to-organ movement (Shitan and Yazaki, 2007; Nour-Eldin and Halkier, 2013)—and is coordinated through a complex network of pathways, including transmembrane transporter–mediated efflux and influx, symplasmic trafficking via plasmodesmata, and vesicle-mediated endocytosis and exocytosis (Verma et al., 2012; Shitan et al., 2014a). Membrane transporters export SMs from source cells into the apoplast or import them into target cells for storage or utilization (Yazaki et al., 2008). These transporters sense concentration changes and regulate dynamic SM levels for accumulation, partitioning, or activation. Manipulating transporter activity has therefore emerged as a powerful strategy for enhancing the accumulation of beneficial metabolites or reducing harmful ones (Zhao, 2015; Liu et al., 2023). Researchers are also developing multifunctional crops by engineering long-distance SM transport systems (Nambiar et al., 2021; Mann et al., 2023). Despite this progress, many aspects remain unresolved, including transporter localization, substrate specificity, affinity, regulation, and coordination of SM transporters in source and sink physiology, particularly in long-distance organ-to-organ transport.

Several studies have identified transporters involved in the spatial distribution of defense and anti-nutritional SMs (Table 1), providing valuable guidance for transporter engineering to enhance desirable SMs or limit the accumulation of undesirable compounds in edible tissues. In this review, we summarize the properties of reported transporters for plant defense and anti-nutritional SMs, focusing on source–sink transporters as engineering targets for crop improvement. Specifically, we address the following questions: (1) How are defense and anti-nutritional compounds transported in plants? (2) Which transporters are involved in SM transport? (3) How can SM transporters be harnessed for crop improvement? (4) What technologies hold promise for characterizing long-distance SM transporters and breeding multifunctional crops? Finally, we discuss challenges that arise in manipulating transporters for crop improvement and potential solutions.

TRANSPORT OF DEFENSE AND ANTI-NUTRITIONAL SECONDARY METABOLITES IN PLANTS

The partitioning of SM synthesis and storage into distinct organelles, cells, and organs is widespread in plants. Lipophilic precursors or intermediates of SMs are typically transported at the intracellular or intercellular level (here referred to as short-distance transport) to balance defensive functions while minimizing autotoxicity. In contrast, hydrophilic SMs such as alkaloids, GSLs, cyanogenic glucosides, iridoid glycosides, and other glycosides are transported across organs via the vascular system (here referred to as long-distance transport). Notably, long-distance transport between source and sink organs through the vascular system is closely integrated with intracellular and intercellular transport processes within those organs (Figure 1). Understanding both the short- and long-distance transport of defensive and anti-nutritional SMs is essential for elucidating

the mechanisms of SM transport and for enabling precise manipulation of transporters in crop improvement.

Short-distance intracellular and intercellular transport of defense and anti-nutritional secondary metabolites

The relocation of metabolites among cellular compartments adds a spatial dimension to plant metabolism and provides an effective mechanism to mitigate self-toxic substances (Shitan, 2016). Intracellular and intercellular transport occur within individual cells or between neighboring cells, regulating the local accumulation, sequestration, or metabolic flux of SMs. For example, cucurbitacin C (CuC), a bitter and defensive triterpenoid, is synthesized and transported within leaf mesophyll cells in cucumber (*Cucumis sativus*) (Shang et al., 2014; Ma et al., 2023). Steroidal glycoalkaloids (SGAs) are transported intracellularly to control toxicity levels in tomato (*Solanum lycopersicum*) fruit (Kazachkova et al., 2021). The predominant and bitter SGA α-tomatine is sequestered into the vacuoles of immature green fruits and later relocated to the cytosol, where it is metabolized into the non-bitter compound esculeoside A during ripening (Kazachkova et al., 2021). This conversion reduces bitterness and toxicity, rendering the fruit safe for consumption. In the medicinal plant *Catharanthus roseus*, the monoterpene indole alkaloid (MIA) pathway is localized across multiple organelles and at least four cell types (St-Pierre et al., 1999; Mahroug et al., 2007). MIA precursorsare synthesized in the cytoplasm and transported into the vacuoles of the leaf epidermis, where biosynthesis of the central intermediate strictosidine and its efflux to the cytosol occur (Payne et al., 2017). This organellar separation likely prevents the accumulation of harmful intermediates. Another well-characterized MIA pathway involves the efflux of catharanthine (an intermediate derived from strictosidine aglycone) from the leaf epidermis to the cuticle (Roepke et al., 2010). Intracellular and intercellular transport pathways involve membrane-transporter-mediated vacuolar sequestration and release, export from the cytosol to the apoplast, vesicle-mediated membrane transport, and symplastic or apoplastic movement between adjacent cells (Halkier and Xu, 2022).

Long-distance source–sink transport of defense and anti-nutritional secondary metabolites

Long-distance transport of SMs involves the translocation of defensive and anti-nutritional metabolites from source organs (e.g., leaves, stems, pods, and roots) to sink organs (e.g., rhizomes, flowers, fruits, and seeds). Efflux from source to sink organs occurs via two main routes: the xylem and the phloem (Figure 1) (Nour-Eldin and Halkier, 2013). The xylem primarily mediates upward transport from roots to shoots, whereas the phloem distributes metabolites bidirectionally between source and sink organs. For instance, berberine, an antibacterial alkaloid, is synthesized mainly in the lateral roots of *Coptis japonica* and transported through the xylem to the rhizome (Ikezawa et al., 2003; Shitan et al., 2003; 2013). Similarly, nicotine, a well-known alkaloid, is synthesized in the roots of tobacco (*Nicotiana tabacum*) and translocated upward through the xylem to aerial tissues, where it accumulates to deter herbivores (Baldwin, 1989, 2001). In *Arabidopsis thaliana* and other Brassicaceae species, GSLs are synthesized primarily in leaves and pods and transported to seeds via long-distance transport in the phloem (Andersen et al., 2013; Xu et al., 2023). In *Arabidopsis*, aliphatic GSLs move bidirectionally between rosettes and roots through the phloem and xylem (Andersen et al., 2013). Rosettes serve as the main source and reservoir for short-chain aliphatic GSLs, while long-chain aliphatic GSLs are synthesized in both rosettes and roots, with roots acting as the major storage site (Andersen et al., 2013). This spatial division of synthesis and storage indicates tightly regulated bidirectional transport to balance systemic distribution. In cassava (*Manihot esculenta*), cyanogenic glucosides are synthesized in leaves and possibly stems and transported via the phloem to the roots, where they serve as a nitrogen source for amino acid synthesis or are stored in vacuoles to prevent autotoxicity (Jørgensen et al., 2005; Gleadow and Møller, 2014). In narrow-leafed lupin (*Lupinus angustifolius* L.), most quinolizidine alkaloids in seeds are translocated from vegetative tissues (leaves, stems, and pods) to seeds through long-distance phloem transport (Lee et al., 2007; Otterbach et al., 2019).

Long-distance source–sink transport involves specialized vascular loading and unloading mechanisms mediated by plasma membrane (PM)-localized transporters, either importers or exporters, in xylem-adjacent parenchyma cells or phloem companion cells (Nour-Eldin and Halkier, 2013). For SMs to enter the vascular stream, they must first be mobilized from their biosynthetic site, usually in the cytosol, and cross membrane barriers to reach the vascular loading cells. In xylem-mediated transport, SMs enter the apoplast near the xylem and are exported into xylem vessels via transporters located on the PM of xylem parenchyma cells, particularly those mediating efflux into the xylem apoplast (Shitan et al., 2003). In phloem-mediated transport, SMs are imported into companion cells across the PM, often via proton-coupled transporters (Nour-Eldin et al., 2012). Once in the phloem, metabolites move through a regulated symplasmic conduit and may undergo retrieval or recycling en route to sink organs (Chen et al., 2001). Upon arrival at sink organs, SMs are unloaded from the vasculature and further transported across membranes or between cells to reach their destination, such as storage vacuoles, epidermal cells, or other defense-related target sites (Sanden et al., 2024).

TRANSPORTERS OF DEFENSE AND ANTI-NUTRITIONAL SECONDARY METABOLITES IN PLANTS

Various mechanisms contribute to the transport and distribution of plant metabolites, including simple diffusion, symplasmic transport through plasmodesmata, vesicle-mediated transport, and substrate-specific membrane transport (Nogia and Pati, 2021). Among these, membrane transporters have emerged as key regulatory nodes and major research targets because of their central roles as gatekeepers of metabolite flux and their potential for genetic and biotechnological manipulation (Lv et al., 2016). These SM transporters are primarily localized to the tonoplast or PM of source and sink cells, as well as to vascular tissues (Figure 2). The main transporter protein families reported include the ATP-binding cassette (ABC) family (Yazaki et al., 2001; Wang et al., 2021), the multidrug and toxic

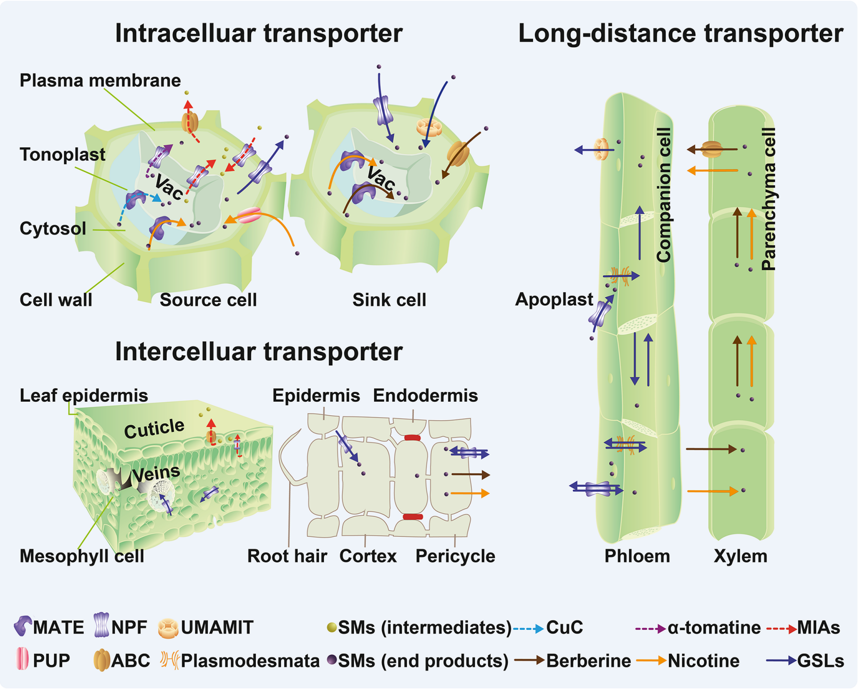

Figure 2. Schematic representation of identified transporters of plant defense and anti-nutritional secondary metabolites.
In source cells, NPF and MATE transporters mediate the movement of secondary metabolites (SMs) between the vacuole and cytosol, while ABC, NPF, and PUP transporters facilitate plasma membrane (PM) efflux and uptake at the cellular interface. In sink cells, MATE transporters import SMs into vacuoles for storage, and NPF, UMAMIT, and ABC family members transport SMs across the PM into the cytosol. Intercellular transporters, including NPF and ABC proteins, mediate SM movement between neighboring cells in leaves and roots. Roots act as the source for berberine and nicotine and as one of the sink organs for glucosinolates. Long-distance transport of these three SMs is mediated by xylem and phloem loading and unloading with the help of NPF, UMAMIT, and ABC transporters. Arrows indicate transport dynamics. Vac, vacuole; CuC, cucurbitacin C; MIAs, monoterpene indole alkaloids; GSLs, glucosinolates.

compound extrusion (MATE) family (Shoji et al., 2009; Ma et al., 2023), the nitrate/peptide transporter family (NPF) (Nour-Eldin et al., 2012; Payne et al., 2017), the purine uptake permease (PUP) family (Hildreth et al., 2011; Dastmalchi et al., 2019), and the recently identified "usually multiple amino acids move in and out transporters" (UMAMIT) (Xu et al., 2023; Sanden et al., 2024) (Table 1). To better illustrate their physiological roles, these transporters can be grouped into intracellular or intercellular transporters (e.g., vacuolar-localized) and source–sink long-distance transporters (e.g., vasculature-localized).

Intracellular and intercellular transporters

Intracellular transporters mediate the import and export of SMs across compartmental boundaries, which is essential for homeostasis, detoxification, and compartmentalized biosynthesis. Tonoplast-localized transporters sequester toxic or storage-bound SMs into vacuoles, while others facilitate remobilization back into the cytosol when metabolites are needed for downstream modification or export. Several members of the MATE and NPF families are located on the tonoplast and act as SM importers or exporters of vacuoles (Figure 2). For example, in cucumber, CsMATE1 is involved in the import of CuC into vacuoles, with its expression co-regulated with CuC biosynthetic genes and transcription factors that directly activate its promoter (Ma et al., 2023). The coordinated biosynthesis and intracellular transport of bitter cucurbitacins may represent a mechanism to regulate the production and spatial partitioning of defensive metabolites while minimizing self-toxicity. In tomato, GORKY from the NPF family is essential for transporting bitter α-tomatine from vacuoles to the cytosol for conversion into the non-bitter esculeoside A (Kazachkova et al., 2021). In *C. roseus*, CrNPF2.9 functions as a vacuolar exporter of strictosidine into the cytosol for downstream modification (Payne et al., 2017).

Transporters at the PM regulate short-distance intercellular movement, either by exporting metabolites to the apoplast or importing them back into the symplasm for further conversion along a biosynthetic pathway, or by transferring them to adjacent storage cells (Halkier and Xu, 2022). This process involves transporters from the ABC and NPF families. For example, the PM-localized ABCG transporter CrTPT2, whose mRNA is predominantly detected in the leaf epidermis, functions as a catharanthine exporter from the epidermis to the leaf surface (Yu and De Luca, 2013). Three other PM-localized CrNPF2.4/CrNPF2.5/CrNPF2.6 transporters of iridoid glucosides, whose genes are expressed in the stem and leaf, are essential for transporting iridoid glucosides (precursors of MIAs) into epidermal cells (Larsen et al., 2017).

Source–sink long-distance transporters

Source–sink long-distance transporters function at the interface of vascular tissues, enabling the mobilization of metabolites from source to sink organs via the xylem and/or phloem. These transporters contribute to SM allocation by regulating cellular uptake or efflux across membranes during long-distance translocation rather than directly partitioning or distributing SMs over long distances (Atkins and Smith 2007; Andersen et al., 2013). They are often localized at the PM of specialized cell types, such as companion cells in the phloem or parenchyma cells adjacent to xylem vessels. They enable the loading and unloading of SMs into transport streams, often using energy-dependent mechanisms such as proton-coupled symport (NPF family) or ATP-driven export (ABC family) (Shitan et al., 2003, 2013; Nour-Eldin et al., 2012) (Figure 2).

Transporters involved in the long-distance transport of GSLs have been well characterized in plants. In *Arabidopsis*, GSL transporters (GTRs) in the NPF and UMAMIT families play key roles in long-distance GSL transport. These transporters

Family	Gene name	Accession no.	Transport substrate	Tissue expression	Subcellular localization	Transport pathway	Species	Reference
ABC	*CjABCB1 (CjMDR1)*	AB043999	berberine	rhizome xylem	PM	uptake in rhizomes	*C. japonica*	Yazaki et al. (2001); Shitan et al. (2003), (2005)
ABC	*CjABCB2*	AB674325	berberine	cells around rhizome xylem	PM	unloading from xylem	*C. japonica*	Shitan et al. (2013)
ABC	*CjABCB3*	AB674326	berberine	–	–	–	*C. japonica*	Shitan et al. (2013)
ABC	*CrTPT2*	KC511771	catharanthine	leaf epidermis	PM	from epidermis to leaf surface	*C. roseus*	Yu and De Luca (2013)
ABC	*LaABCB11*	–	lycorine	phloem of leaf, bulb, and root	PM	from leaves and bulbs to roots	*L. aurea*	Wang et al. (2021)
ABC	*AmABCB1*	UFQ90028	sanguinarine, berberine	mature seed, pericarp	PM	–	*A. mexicana*	Loza-Muller et al. (2021)
MATE	*NtMATE1*	AB286961	nicotine, hyoscyamine, scopolamine	root	tonoplast	from cytosol to vacuole	*N. tabacum*	Shoji et al. (2009)
MATE	*NtMATE2*	AB286962	nicotine	root	tonoplast	from cytosol to vacuole	*N. tabacum*	Shoji et al. (2009)
MATE	*NtJAT1*	AM991692	nicotine, berberine	leaf, stem, root	tonoplast	sequestration in leaf vacuole	*N. tabacum*	Morita et al. (2009); Yamada et al. (2022)
MATE	*NtJAT2*	AB922128	nicotine, berberine	leaf	tonoplast	sequestration in leaf vacuole	*N. tabacum*	Shitan et al. (2014b)
MATE	*CjMATE1*	LC199487	berberine	rhizome	tonoplast	stored in vacuole	*C. japonica*	Takanashi et al. (2017)
MATE	*CsMATE1*	Csa1G044870	cucurbitacin C	leaf, stem, fruit	tonoplast	from cytosol to vacuole	*C. sativus*	Ma et al. (2023)
MATE	–	Manes. 16G007900/Manes. 16G008000	cyanogenic glucosides	root and shoot apical meristems	–	–	*M. esculenta*	Ogbonna et al. (2021)
NPF	*CrNPF2.9*	KX372303	strictosidine	leaf epidermis	tonoplast	from vacuole into cytosol	*C. roseus*	Payne et al. (2017)
NPF	*CrNPF2.4/ CrNPF2.5/ CrNPF2.6*	ALE20039/ ALE20040/ ALE20041	iridoid glucosides	stem, leaf	PM	into epidermal cells	*C. roseus*	Larsen et al. (2017)
NPF	*GORKY*	Solyc03g120570	α-tomatine	fruit	tonoplast	from vacuole to cytosol	*S. lycopersicum*	Kazachkova et al. (2021)
NPF	*MeCGTR1*	Me15g18400	linamarin	–	–	–	*M. esculenta*	Jørgensen et al. (2017)
NPF	*GTR1*	AT3G47960	glucosinolates	vasculature, mesophyll cells, root cortex	PM	seed loading, movement between cells	*Arabidopsis thaliana*	Nour-Eldin et al. (2012)

Table 1. Transporters involved in the transport of defense and anti-nutritional secondary metabolites in plants.

(Continued on next page)

Family	Gene name	Accession no.	Transport substrate	Tissue expression	Subcellular localization	Transport pathway	Species	Reference
NPF	*GTR2*	AT5G62680	glucosinolates	veins	PM	seed loading, import into phloem	*A. thaliana*	Nour-Eldin et al. (2012)
NPF	*GTR3*	AT1G18880	indole glucosinolates	root phloem	–	retained in roots	*A. thaliana*	Jørgensen et al. (2017)
NPF	*BjuGTR1/ BjuGTR2*	–	glucosinolates	seed, pod wall	–	from siliques to seeds	*B. juncea*	Nour-Eldin et al. (2017); Nambiar et al. (2021); Mann et al. (2023)
NPF	*BnaC02.GTR2*	BnaC02g42260D	glucosinolates	–	–	from siliques to seeds	*B. napus*	Tan et al. (2022)
NPF	*BnaA06.GTR2*	–	glucosinolates	silique wall	–	from siliques to seeds	*B. napus*	He et al. (2022)
PUP	*NtNUP1*	GU174267	nicotine, vitamin B6	leaf, root (especially root tip)	PM	from apoplast to cytosol	*N. tabacum*	Hildreth et al. (2011); Kato et al. (2015)
PUP	*PUP1*	–	tropane	–	tonoplast	–	*A. belladonna*	Srinivasan and Smolke (2021)
PUP	*CsPUP10.1*	–	caffeine	–	PM	–	*C. sinensis*	Zhang et al. (2022)
PUP	*BUP1*	MH838003	benzylisoquinoline	latex	PM	–	*Papaver somniferum*	Dastmalchi et al. (2019)
UMAMIT	*UMAMIT29*	AT4G01430	glucosinolates	funiculi vasculature, biosynthetic cells, chalazal seed coat	PM	from siliques to seeds	*A. thaliana*	Xu et al. (2023); Sanden et al. (2024)
UMAMIT	*UMAMIT30/31*	AT4G01440/ AT4G01450	glucosinolates	funiculi	PM	from siliques to seeds	*A. thaliana*	Xu et al. (2023); Sanden et al. (2024)

Table 1. *Continued*
PM, plasma membrane.

are essential for various processes, including GSL redistribution within leaves, exudation from roots, seed loading, and directed movement to stem S-cells for storage (Nour-Eldin et al., 2012; Madsen et al., 2014; Xu et al., 2017; 2019). They facilitate GSL trafficking among roots, shoots, leaves, pods, and seeds (Hunziker et al., 2019, 2021). In leaves, GTR1 and GTR2 function as PM-localized transporters, primarily expressed in veins, while *GTR1* is also expressed in adjacent mesophyll cells (Nour-Eldin et al., 2012). GSLs are transported across the PM into phloem companion cells in leaves via GTR1 and GTR2, with GTR1 also mediating import into mesophyll cells. In roots, GTR1 and GTR2 are predominantly localized to cortex and vascular cells, contributing to the distribution of indole GSLs between root and shoot (Jørgensen et al., 2017; Xu et al., 2017). *GTR3* is strongly expressed in the companion cells of the root phloem, where it facilitates the retention of indole GSLs in roots (Jørgensen et al., 2017). Additionally, three PM-localized UMAMITs (UMAMIT29, UMAMIT30, and UMAMIT31) are involved in GSL movement from siliques to seeds via funiculus export (Xu et al., 2023). UMAMIT29 is localized in cortex cells and cells adjacent to xylem vessels in the funiculus, as well as in the outer integument and chalazal seed coat. UMAMITs also export GSLs from biosynthetic cells to the apoplast surrounding the vasculature (Sanden et al., 2024).

Intracellular and intercellular transporters involved in long-distance translocation

Some transporters indirectly support long-distance SM movements, acting through intracellular and intercellular transport rather than vascular pathways. For example, the tonoplast-localized *CjMATE1*—predominantly expressed in rhizomes but also in leaves, petioles, and roots—encodes a protein that facilitates berberine accumulation in vacuoles (Takanashi et al., 2017). Similarly, nicotine transporters NtMATE1 and NtMATE2 are abundant in root tissues, encoding tonoplast proteins that mediate nicotine transport from the cytosol to vacuoles (Shoji et al., 2009). The PM-localized NtNUP1 assists in the movement of apoplastic nicotine into the cytoplasm of root cells (Hildreth et al., 2011). Additionally, tonoplast-localized NtJAT1 and NtJAT2 are responsible for nicotine sequestration in leaf vacuoles (Morita et al., 2009; Shitan et al., 2014b). Although *NtJAT1* is detected in leaves, stems, and roots, *NtJAT2* is expressed specifically in leaves. The expression patterns and localization of these two transporters do not support a role in unloading nicotine from the xylem into leaf cells. Certain membrane transporters, such as GTR1, participate in both long-distance translocation via the vascular system and intercellular transport near the xylem or phloem, reflecting the interconnection between local metabolism and systemic metabolite distribution (Madsen et al., 2014).

MANIPULATING TRANSPORTERS FOR CROP IMPROVEMENT

The structural and functional diversity of SMs means that modifying their transport can affect multiple processes in plants. Manipulating SM transporters could regulate metabolite production, direct their accumulation to specific tissues or organs, and enhance resistance to biotic and abiotic stresses, while reducing anti-nutritional compounds in edible parts (Nogia and Pati, 2021). In the following sections, we explore the potential of transporter engineering to influence intracellular and intercellular transport (e.g., catharanthine, glycoalkaloids, and cucurbitacins) and long-distance translocation (e.g., berberine, nicotine, and GSLs) of SMs in plants (Table 2).

Engineering of defense and bitter secondary metabolite transporters involved in intracellular or intercellular processes

Engineering transporters involved in intracellular and intercellular transport has emerged as a useful strategy for crop improvement by modulating the accumulation of bioactive compounds in plants. These transporters often affect the accumulation of SMs in specific organelles or cells. By controlling the expression of these transporters, plants may alter the yield of end products by regulating metabolic flux toward intermediates or final products. Several studies illustrate this potential. In cucumber, *CsMATE1* knockout mutants showed a significant decrease in the accumulation of bitter CuC in cotyledons (Ma et al., 2023). In tomato, *GORKY* overexpression decreased bitter α-tomatine and increased non-bitter esculeoside A in leaves and fruits (Kazachkova et al., 2021). The success of reducing bitterness to improve crop traits depends on a detailed understanding of transporter function and SM flux at the organelle level. Multiple genetic approaches support roles for these transporters as metabolite valves on the tonoplast. In cucumber, *CsMATE1* knockout reduced vacuolar CuC content by 65%–70% and suppressed the expression of CuC biosynthesis genes, confirming its role as a vacuolar importer and revealing feedback between transport and biosynthesis (Ma et al., 2023). Silencing *GORKY* elevated α-tomatine and reduced esculeoside A in infected tomato fruit, while knockout mutants accumulated upstream SGA intermediates and depleted the final non-bitter product (Kazachkova et al., 2021).

This strategy also applies to defense or pharmaceutically relevant alkaloids such as MIAs. In *C. roseus, CrTPT2* knockdown reduced catharanthine levels on the leaf surface but increased it within leaves (Yu and De Luca, 2013). Conversely, *CrTPT2* overexpression resulted in a fivefold increase in catharanthine in hairy roots (Wang et al., 2019). Silencing *CrNPF2.9* caused a strong increase in strictosidine together with a decrease in MIA end products, consistent with the role of CrNPF2.9 as a vacuolar exporter (Payne et al., 2017). Overexpression could potentially be used to increase MIA accumulation in *C. roseus*.

These examples collectively highlight that manipulating intracellular and intercellular transporters can reduce the accumulation of toxic intermediates or undesirable bitter compounds while increasing yields of pharmacologically important SMs. To minimize potential risks associated with transporter engineering, such as pleiotropic developmental defects observed in *GORKY*-overexpressing tomato (e.g., dwarfism and floral deformities) and *CrNPF2*.9-silenced *C. roseus* (leaf cell death), tissue-specific promoters or backcrossing into elite lines could be employed. Furthermore, the physiological roles of some SMs pose trade-offs that must be considered in crop breeding.

Secondary metabolites	Targets	Engineered method	Engineered results	Crop improvement outcome	Reference
Cucurbitacin C 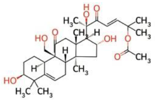	*CsMATE1*	CRISPR/Cas9	Significantly decreased CuC levels in vacuoles	Reduced bitterness in plants	Ma et al., 2023
		Overexpression	Significantly increased CuC levels in cotyledons	Not reported	
α-tomatine 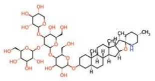	*GORKY*	Overexpression	Decreased α-tomatine and increased esculeoside A in leaves and fruits	Reduced bitterness in fruits	Kazachkova et al., 2021
		VIGS, CRISPR/Cas9	Increased α-tomatine and decreased esculeoside A in fruits	Not reported	
Strictosidine 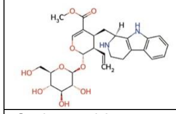	*CrNPF2.9*	VIGS	Increased strictosidine and decreased MIAs in leaves	Not reported	Payne et al., 2017
Catharanthine 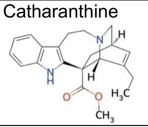	*CrTPT2*	VIGS	Decreased catharanthine on leaf surface and increased within leaves	Commercially useful plants with high anticancer alkaloids	Yu and De Luca, 2013
		Overexpression	5-fold increase of catharanthine in hairy roots	Alkaloid production in heterologous	Wang et al., 2019
Berberine 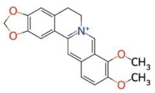	*CjABCB1 (CjMDR1)*	Rhizobium-mediated co-suppression	Decreased berberine in leaves, petioles, and roots	No improvement observed	Shitan et al., 2005
Nicotine	*NtMATE1*	RNAi	No change in leaves and roots	No improvement observed	Shoji et al., 2009
	NtMATE2	RNAi	No change in leaves and roots	No improvement observed	
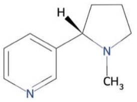	*NtNUP1*	RNAi	Decreased nicotine in hairy roots but increased in culture medium; decreased in roots and leaves	Reduced alkaloid content in plants with improved root growth	Hildreth et al., 2011; Kato et al., 2015"
	Nt-JAT1	Expressed in *E.coli*	14-fold increase of nicotine	Alkaloid production in heterologous system	Yamada et al., 2022
Indole glucosinolates 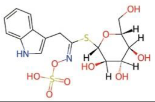	*GTR3*	CRISPR/Cas9	Lower indole GSLs in roots but elevated levels in rosettes	Not reported	Jørgensen et al., 2017
	GTR1/GTR2	CRISPR/Cas9	Increased indole GSLs in rosettes	Not reported	
Glucosinolates 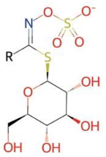	*GTR1*	CRISPR/Cas9	No change in seed GSLs	Not reported	Nour-Eldin et al., 2012
	GTR2	CRISPR/Cas9	48 ± 11% reduction of GSLs in seeds	Not reported	
	GTR1/GTR2	CRISPR/Cas9	Undetectable seed GSLs; 10-fold over-accumulation in leaves and silique walls	Not reported	
	UMAMIT29	CRISPR/Cas9	80% reduction of GSLs in seeds; comparable GSLs in siliques	Not reported	Xu et al., 2023
	UMAMIT29/30/31	CRISPR/Cas9	95% reduction of GSLs in seeds	Not reported	
	Four *BjuGTR2s*	TILLING	62±10% reduction of seed GSLs (44±11 μM/g)	Low GSL content in seeds	Nour-Eldin et al., 2017
		RNAi	>50% reduction of seed GSLs (30.06 μM/g); increased GSLs in leaves and pods	Edible seeds with enhanced pest resistance	Nambiar et al., 2021
	BjuGTR1	RNAi	Decreased GSLs in seeds, pods and leaves	No improvement observed	
	BjuGTR1/2	RNAi	Decreased GSLs in seeds, pods and leaves	No improvement observed	
	BnaC02.GTR2	CRISPR/Cas9	86.85% reduction of seed GSLs (18.21 μM/g); decreased leaf GSLs	Edible seeds but reduced seed quality	Tan et al., 2022
	BnaA06.GTR2	CRISPR/Cas9	76.05% reduction of seed GSLs (30 μM/g); low GSL levels in silique walls	Edible seeds with normal morphology or 1000-seed weight	He et al., 2022
	multiple *BjuGTR1* and *BjuGTR2*	CRISPR/Cas9	Decreased seed GSLs (<30 μM/g); high leaf GSLs (>70 μM/g); high pod GSLs (30–70 μM/g)	Edible seeds with uncompromised defense response and unaltered seed quality	Mann et al., 2023

Table 2. Engineered transporters involved in intracellular (blue), intercellular (orange) and long-distance organ-to-organ (green) transport of defensive and bitter secondary metabolites for crop improvement.
CuC, cucurbitacin C; MIAs, monoterpene indole alkaloids; GSLs, glucosinolates.

For instance, CuC imparts bitterness to edible tissues but also contributes to plant defense. However, it remains unclear whether all CuC in cucumber fruits is synthesized locally or partly transported from leaves, and CuC levels in *CsMATE1* mutant fruits have yet to be analyzed (Shang et al., 2014; Ma et al., 2023).

Manipulation of transporters that mediate long-distance organ-to-organ transport

Using transporters that mediate long-distance organ-to-organ transport to regulate the distribution of defense and bitter SMs remains relatively underexplored. In tobacco, silencing of *NtMATE1/2* through RNA interference (RNAi) failed to alter nicotine profiles in roots and leaves (Shoji et al., 2009). Downregulation of *NtNUP1* by RNAi decreased nicotine accumulation in roots and leaves without affecting root-to-shoot translocation (Hildreth et al., 2011). These findings suggest that although certain transporters mediate the movement of SMs that are ultimately distributed via long-distance pathways, their direct roles may be restricted to cellular uptake, intracellular sequestration, or local export within source or sink tissues rather than vascular loading or unloading.

Further functional characterization revealed that nicotine transporters are not strictly nicotine specific but exhibit broad substrate specificity. For example, several of these transporters efficiently transported hyoscyamine, NtJAT1 and NtJAT2 transported berberine, and NtNUP1 also transported vitamin B6 (Kato et al., 2015). Such substrate promiscuity poses a challenge for targeted engineering and suggests a need for context-dependent validation of transporter functions. Although heterologous expression of *NtJAT1* in *Escherichia coli* increased alkaloid production by 14-fold, its *in planta* function remains unclear, raising questions about interaction with endogenous transport systems and underscoring the complexity of engineering SM transporters (Yamada et al., 2022). The limited success in manipulating such transporters further underscores the need to identify and characterize long-distance transporters that could influence source–sink efflux. For example, manipulation of PM-localized CjABCB1 or CjABCB2 may affect root-to-rhizome berberine translocation in *C. japonica*, as both are highly expressed in rhizome xylem and have been implicated in berberine uptake (Shitan et al., 2003; 2013). However, in transgenic plants with co-suppressed *CjABCB1*, berberine levels were unexpectedly reduced in leaves and petioles (non-primary accumulation sites) as well as in roots (source organ) (Shitan et al., 2005). Due to the lack of expression and metabolite data from the rhizome (sink organ), it remains unclear whether xylem unloading is altered in this tissue.

Inhibition of long-distance transport produces “low-glucosinolate” seeds with defense potential

Following advances in engineering short-distance SM transporters, the potential for harnessing long-distance transporters is becoming increasingly evident. Also, the historical reduction of bitterness in plants may have overlooked the value of these compounds (Liu et al., 2022). Emerging research underscores that bitter SMs such as GSLs and alkaloids not only defend plants against pests and pathogens but also possess therapeutic potential for humans, including anti-inflammatory, antioxidant, and antitumor activities (Drewnowski and Gomez-Carneros, 2000; Liu et al., 2022). Given their dual role as plant protectors and nutraceuticals, engineering SM long-distance transporters should focus on retaining their benefits in vegetative tissues while minimizing their presence in edible organs.

By targeting GSL long-distance transporters such as GTR1, GTR2, GTR3, or UMAMIT29/30/31, researchers can block GSL loading into seeds while maintaining biosynthesis and storage in leaves (Kumar et al., 2017; Liu et al., 2020). In *Arabidopsis*, knockout mutants of *GTR1* and *GTR2* show distinct roles in GSL allocation: *gtr1* mutants retain wild-type seed GSL levels, whereas *gtr2* mutants display a significant reduction (48% ± 11%) (Nour-Eldin et al., 2012). The *gtr1 gtr2* double mutant eliminates detectable seed GSLs, highlighting the dominant role of GTR2 in seed loading and a compensatory role for GTR1. This may be linked to GTR1’s additional role in mesophyll cells. In *gtr3* mutants, indole GSLs are significantly lower in roots but elevated in the rosette. Furthermore, the *gtr1 gtr2* double mutant showed increased rosette indole GSLs, which was fourfold that of the *GTR3* knockout mutant (Jørgensen et al., 2017). Beyond the GTR family, *UMAMIT29/30/31* transporters are highly expressed in funiculi—the only vascular connections between the silique septum and the seed (Xu et al., 2023; Sanden et al., 2024). Mutant analysis shows comparable silique GSLs but an 80% reduction in seed GSLs in *umamit29* and less than 5% of seed GSLs in the *umamit29 umamit30 umamit31* triple mutant compared with the wild type, demonstrating nonredundant functions in seed loading (Xu et al., 2023). The divergent outcomes of manipulating these transporters, from tissue-specific redistribution (e.g., GTR3) to near-complete elimination of seed GSLs (UMAMIT29/30/31), reflect distinct biochemical roles, spatial expression, and compensation. Although manipulating these transporters alters GSL efflux from source to sink organs, the impact of increased GSLs in the source on plant defense has not been specifically addressed.

In *Brassica juncea* and *Brassica napus*, research on GTR manipulation has focused on multiple *GTR1* and *GTR2* homologs. In *B. juncea*, four functional *BjuGTR2* homologs were analyzed by TILLING (Nour-Eldin et al., 2017). Quadruple mutations in *BjuGTR2* led to a 62% ± 10% reduction in seed GSL content compared to the wild type, with a GSL concentration of 44 ± 11 μM/g dry weight (DW), exceeding the canola standard of 30 μM/g DW (Nour-Eldin et al., 2017). RNAi-mediated knockdown of *BjuGTR2* resulted in a >50% reduction in seed GSL levels (30.06 μM/g DW) while simultaneously increasing GSL concentrations in the leaves and pods compared with wild-type plants, enhancing resistance against generalist pests (Nambiar et al., 2021). However, *BjuGTR1*-silenced and *BjuGTR1/GTR2*-co-silenced lines showed reduced GSL levels in seeds, leaves, and pods, compromising defense against herbivores (Nambiar et al., 2021). In *B. napus*, Tan et al. (2022) mutated *GTR2* homologs using CRISPR–Cas9-based editing. Association mapping identified *BnaC02.GTR2* as the key homolog regulating seed GSL transport. Editing *BnaC02.GTR2* together with three other *GTR2* homologs reduced seed GSLs by 86.85% to 18.21 μM/g (Tan et al., 2022). These edits also reduced leaf GSL content, 1000-seed weight, and seed size, and altered seed amino acid, sugar, and fatty acid composition (Tan et al., 2022). In contrast, a loss-of-function mutation in *BnaA06.GTR2* reduced seed GSLs by 76.05% to approximately 30 μM/g (He et al., 2022). Notably, these mutants exhibited no apparent changes in morphology or 1000-seed weight but lower-than-normal GSL levels in developing silique walls (He et al., 2022). Recently, simultaneous mutation of multiple *BjuGTR1* and *BjuGTR2* homologs in *B. juncea* using CRISPR–Cas9 reduced seed GSL to <30 μM/g DW in T2 and T3 plants, with high leaf GSL content (>70 μM/g DW) and relatively high pod GSLs

(30–70 μM/g DW) (Mann et al., 2023). Despite these changes, the mutants maintained defense response and seed quality, underscoring the promise of modifying SM long-distance transporters for crop improvement.

Research on GSL transport in *Arabidopsis* and *Brassica* provides critical insight into how SMs, such as GSLs, are mobilized and highlights the importance of understanding transport mechanisms for breeding. By manipulating long-distance transporters responsible for vascular loading and unloading and relocation in source and sink organs, it may be possible to improve seed quality while maintaining effective defense. Key factors for success include identifying specific transporters and selecting appropriate editing strategies. For example, GTR1 functions as a compensatory and intercellular transporter (Nour-Eldin et al., 2012). Thus, editing *GTR2* and *BjuGTR2* rather than *GTR1* and *BjuGTR1* has been more effective for reducing seed GSLs while preserving defense in source tissues (Nambiar et al., 2021). Additional considerations contribute to the successful manipulation of SM transport. These include source–sink relationships, transport directionality, subcellular localization, specific cell types in which transporters act, and whether they serve as gatekeepers of long-distance vascular transport (e.g., phloem or xylem loading). Substrate specificity also affects selectivity and feasibility; for instance, GTR1 contributes to jasmonate transport in *Arabidopsis*, which is important for stress signaling (Ishimaru et al., 2017). Therefore, engineering strategies targeting GTR1 may cause unintended effects, such as reduced stress tolerance. In species with multiple homologs, such as *Brassica*, decisions regarding which transporters to edit—whether individually or in combination—as well as the choice of gene-editing method and target sites, can profoundly influence the phenotype, sometimes leading to adverse effects. Therefore, breeding strategies should undergo iterative optimization to achieve the desired crop improvements. For example, because *GTR2* has multiple homologs in *Brassicaceae*, selecting a homolog with a suitable expression pattern is important (Mann et al., 2023). He et al. (2022) edited *BnaA06.GTR2*, whose expression mirrors the GSL accumulation pattern, to mitigate the adverse effects on morphology and 1000-seed weight observed in multi-homolog mutations in *B. napus*.

TECHNOLOGIES AND FRAMEWORKS FOR DISCOVERING AND CHARACTERIZING LONG-DISTANCE SECONDARY METABOLITE TRANSPORTERS FOR CROP IMPROVEMENT

Manipulating intra- and intercellular transporters involved in both short-distance and long-distance SM transport has clarified transporter functions and underscored the importance of harnessing SM transporters in crop improvement. Compared with intra- and intercellular transporters, long-distance transporters hold greater potential for breeding multifunctional crops that enhance defense while minimizing harmful or anti-nutritional compounds in seeds. It then becomes possible to manipulate transporters to achieve specific crop improvement goals with precision. In the following sections, we explore the technologies and frameworks for the discovery and characterization of long-distance transporters of SMs for crop improvement (Figure 3; Table 3).

Discover long-distance transport pathways and related transporters

Building on the successful examples discussed above, the primary question is whether SMs are synthesized *in situ* or transported from other tissues. Several methods can be employed to confirm the existence of long-distance SM transport and clarify source–sink relationships (Table 3). One widely used strategy is to confirm tissue-specific expression patterns of SM biosynthetic genes to identify the primary site of SM biosynthesis (Shoji et al., 2002). Grafting experiments between SM-producing and non-producing genotypes, combined with sap metabolite profiling, can reveal whether SMs move across graft junctions, providing direct evidence of long-distance transport (Xu et al., 2023). Quantitative analysis of SM intermediates or final products across tissues and in xylem or phloem sap offers insight into transport direction and potential vascular loading (Lee et al., 2007). Metabolic tracing experiments (e.g., isotope or stable-label tracking) can monitor real-time movement of SMs through plant tissues (Chokkathukalam et al., 2014). Together, these techniques determine whether a metabolite is produced and retained locally or redistributed via vascular networks. Confirming the existence, directionality, and tissue specificity of such transport is essential for identifying source and sink organs and represents the first step in designing effective transporter-based strategies for crop improvement.

While long-distance SM transport has been documented in species such as lupins and cassias, identifying and functionally validating candidate transporters remains challenging. In particular, integrating advanced molecular and genetic approaches is essential for the identification of these transporters (Figure 3). Recent studies have applied quantitative trait locus mapping and genome-wide association studies, alone or together with RNA sequencing analysis followed by RT–qPCR validation, to predict and identify potential transporters (Tan et al., 2022). For example, two MATE transporters identified through genome-wide association studies showed differential expression between fibrous and storage roots, highlighting their potential role in the transport of cyanogenic glucosides (Ogbonna et al., 2021). Transcriptomic analysis indicated that *LaABCB11, which* encodes a PM-localized lycorine transporter in *Lycoris aurea*, was primarily expressed in the phloem of leaves, bulbs, and roots (Wang et al., 2021). Co-expression analysis with known biosynthetic pathway genes also helps identify co-regulated transporters involved in metabolite transport (Hildreth et al., 2011; Kato et al., 2015; Jørgensen et al., 2017; Yamada et al., 2022). Using these approaches, Srinivasan and Smolke (2021) identified a tropane alkaloid transporter, PUP1, in *Atropa belladonna*, a vacuolar, tonoplast-associated transporter (Srinivasan and Smolke, 2021). Homology modeling and collinearity analyses of known SM transporters offer a straightforward route to nominate candidates in other crops (Ma et al., 2023). These methods identified CsPUP10.1 in *Camellia sinensis*, suggesting that this PM-localized transporter is involved in caffeine intracellular transport (Zhang et al., 2022). Moreover, AmABCB1, an ortholog of CjABCB1 in *Argemone*

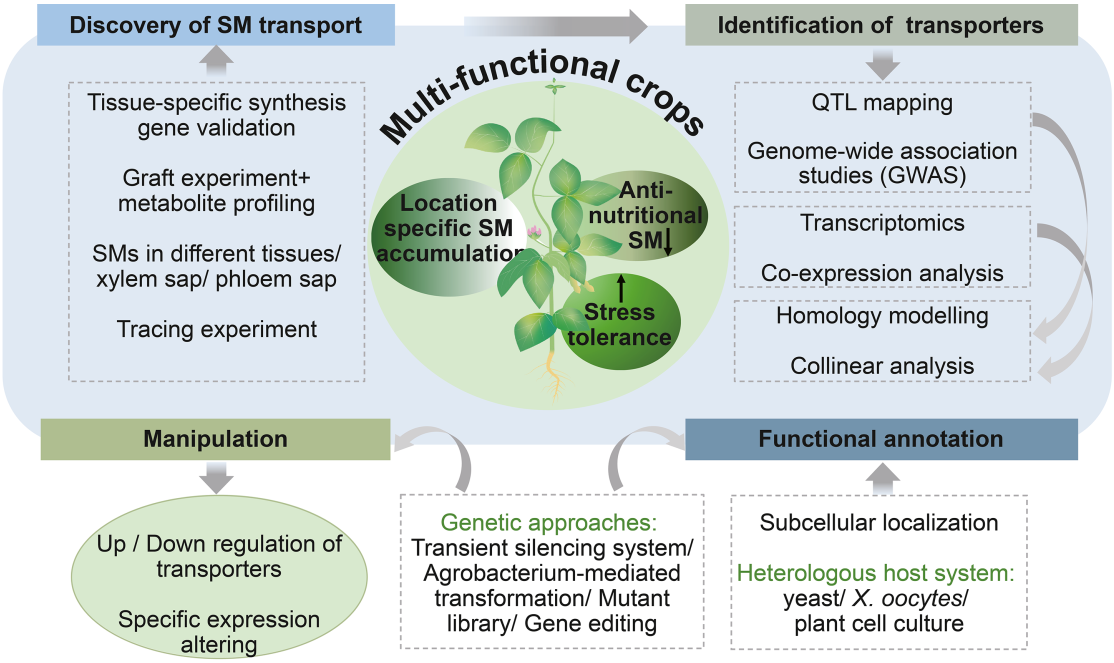

Figure 3. Schematic illustration of approaches for discovering, identifying, and functionally annotating plant secondary-metabolite long distance transporters, and of transporter engineering models and applications for crop improvement.

mexicana, possessed uptake and efflux activities for sanguinarine and berberine in yeast assays (Loza-Muller et al., 2021).

Inferring transporter function through localization analyses

Once candidate SM transporters are identified, subcellular and tissue-level localization analyses provide critical clues to their roles, especially for distinguishing short-distance from long-distance functions. Confocal microscopy with organelle marker fusions defines the targeted membrane or organelle and thus the transporter's mode of action (Payne et al., 2017). High-resolution cell- or tissue-specific expression analysis using β-glucuronidase reporters or *in situ* hybridization reveals where and when the transporter gene is expressed, linking it to developmental processes or key transport barriers (Shoji et al., 2009; Sanden et al., 2024). These approaches can be combined to map transporter activity spatially and generate strong preliminary evidence for physiological roles. The PM is a key site for long-distance transporters because it mediates export to or import from the apoplast (Halkier and Xu, 2022). Particular attention should be given to vascular tissues as metabolic efflux pathways and vascular connections between sinks and sources, such as funiculi (Xu et al., 2023). These localization analyses form a foundation for inferring function and selecting candidates for physiological and genetic validation.

Heterologous expression systems as powerful tools for studying secondary metabolite transporters

Heterologous expression systems such as *Xenopus laevis* oocytes and yeast strains are indispensable for characterizing SM transporter function, including substrate specificity, ion coupling, and transport direction (efflux or uptake). The *X. laevis* oocyte system has been instrumental in identifying the first long-distance GTRs (Nour-Eldin et al., 2012) and in defining substrate specificity under controlled intracellular conditions (Pike et al., 2019). The yeast (*Saccharomyces cerevisiae*) system is widely used because it is easy to manipulate and has low endogenous transport background. When a plant transporter is expressed in yeast, changes in metabolite accumulation can indicate its substrate specificity and the direction of transport (Morita et al., 2009). In addition, plant-based systems such as tobacco BY-2 cells, other suspensions, and protoplasts offer more native-like contexts. For example, GSL uptake assays in cotton cell suspensions (Nambiar et al., 2021) and confocal imaging of transporter activity in mesophyll protoplasts (Hang et al., 2024) have expanded the toolkit. Choice of system should match coupling mechanism. For example, H^+ -driven MATE antiporters can be assayed in oocytes using voltage clamp (Ma et al., 2023). H^+ symport PUP transporters are often better studied in yeast due to stable pH gradients (Hildreth et al., 2011; Dastmalchi et al., 2019), whereas the ATP-driven ABC family transporters can be characterized in both yeast and oocytes (Lefèvre and Boutry, 2018). When the focus is on metabolite compartmentation, plant cell systems can provide more informative insights. Beyond functional studies, heterologous expression, especially in plant cell cultures, supports synthetic biology by enabling reconstitution of transport modules to improve metabolite flux, secretion, or storage in microbial or plant chassis (Wu et al., 2021).

Functional validation of secondary metabolite transporters and genetic manipulation for crop improvement

Experimental validation of SM transporters employs RNAi, overexpression, virus-induced gene silencing, mutant library screening, and CRISPR–Cas9 editing. RNAi and overexpression

Purpose	System/method	Tools and techniques	Functional outcome
Discovery of long-distance SM transport	tissue-specific biosynthetic gene validation	RT–qPCR, GUS reporter assay	identification of SM biosynthesis site
	metabolite profiling	LC–MS, HPLC, GC–MS	SM quantification
	grafting experiments	scion/rootstock combinations	determination of transport direction
	xylem/phloem sap analysis	EDTA-facilitated exudation	direct evidence of transport
	tracing experiments	isotopic labeling, fluorescent tracers	real-time transport monitoring
Identification of transporters	QTL mapping/GWAS	SNP arrays, linkage analysis	locus identification
	transcriptomics	RNA-seq, RT–qPCR	tissue- and stage-specific candidate identification
	homology modeling/collinearity	sequence alignment, phylogenetic analysis	identification of orthologs in non-model species
	co-expression analysis	WGCNA, clustering	co-regulated gene prediction
Functional annotation	subcellular localization	organelle marker fusion, confocal microscopy	determination of membrane localization
	cell-/tissue-specific expression analysis	GUS assay, *in situ* hybridization	resolves of tissue- and cell-level distribution
	X. laevis oocyte system	microinjection, voltage clamp, uptake assays	transporter screening, substrate specificity
	yeast system	mutant complementation, radiolabeled substrates	substrate validation, heterologous SM biosynthesis
	plant cell culture	suspension cultures, protoplasts	uptake analysis in a plant background, heterologous SM biosynthesis
	transient gene silencing system	VIGS	rapid knockdown
Functional annotation and manipulation	*Agrobacterium*-mediated transformation	overexpression, RNAi, tissue-specific promoters	precisely controlled expression
	mutant library	EMS mutagenesis, TILLING	high-throughput mutant screening
	gene editing	CRISPR–Cas9	precise gene knockout or editing

Table 3. Experimental tools and techniques for investigating and modifying long-distance transport of plant secondary metabolites (SMs).

GUS, β-glucuronidase; LC–MS, liquid chromatography–mass spectrometry; HPLC, high-performance liquid chromatography; GC–MS, gas chromatography–mass spectrometry; EDTA, ethylenediaminetetraacetic acid; WGCNA, weighted gene co-expression network analysis; QTL, quantitative trait locus; GWAS, genome-wide association study; SNP, single-nucleotide polymorphism; RNAi, RNA interference; EMS, ethyl methanesulfonate; TILLING, targeting induced local lesions in genomes; VIGS, virus-induced gene silencing; CRISPR–Cas9, clustered regularly interspaced short palindromic repeats–CRISPR-associated protein 9.

are typically performed via *Agrobacterium*-mediated transformation, using *A. tumefaciens* for stable transformation and *A. rhizogenes* for roots (Ricigliano et al., 2016). Transformation efficiency remains a limitation for some crops, such as lupin (Uhde-Stone et al., 2005; Sbabou et al., 2010; Cheng et al., 2011). Virus-induced gene silencing offers faster validation (Mancinotti et al., 2021), but its transient nature poses challenges: source–sink phenotypes must be observed before silencing diminishes, and assessing seed- or fruit-related traits can be difficult because silencing duration may not match reproductive cycles. Shortening the reproductive period and extending silencing duration may help address this issue.

Gene silencing can markedly reduce expression (knockdown) but may not fully eliminate it (Alagoz et al., 2016). Mutant libraries combined with TILLING can yield loss-of-function alleles or new variants for functional studies and trait improvement, but the random, unpredictable nature of such mutants requires labor-intensive, time-consuming screening (Sikora et al., 2011). For more a thorough understanding of gene function and targeted modification of specific metabolites, gene-editing tools such as CRISPR–Cas9 are recommended (Zhang et al., 2021). Optimizing CRISPR technology is essential for accommodating the delivery method in recalcitrant and difficult-to-regenerate species such as lupin (Pigeaire et al., 1997). For example, a model allotetraploid tobacco host was engineered to undergo single, multiplex, and chromosomal deletions at a high frequency using a negative-strand RNA virus-based vector for DNA-free delivery of the entire CRISPR–Cas9 cassette (Ma et al., 2020). Additional delivery methods using functionalized nanoparticles have been developed in cotton, sunflower, and lily, enabling transformation without *in vitro* regeneration (Zhao et al., 2017). Functionalized magnetic particles have further enhanced transformation efficiency, accelerating breeding and

selection (Watson et al., 2018; Lew et al., 2020). Advances in nanoparticle technologies have also increased CRISPR–Cas9 efficiency, enabling transformation in species previously considered difficult (Ma et al., 2021). These genetic approaches not only validate SM transporters but also allow their manipulation for diverse breeding purposes. In particular, subcellular-level phenotypic analysis is required when whole-organ SM quantification cannot resolve transporter functions, especially for proteins mediating compartmentalized transport (e.g., vacuolar importers or exporters). For example, isolating protoplasts and vacuoles for CuC quantification in *CsMATE1* mutants has provided more precise insight into transporter functionality (Ma et al., 2023), highlighting the need for higher-resolution assays to characterize transporters involved in specific subcellular trafficking pathways.

In species such as narrow-leafed lupin, long-distance transport of defense SMs has been documented (Lee et al., 2007; Otterbach et al., 2019), but the specific transporters involved have yet to be identified. While various candidate transporters for defense SMs are proposed in Table 1, their functions require further validation. Using advanced tools, SM transporters can be identified, validated, and modulated through upregulation, downregulation, or targeted expression to enhance metabolite production, improve tissue- or organ-specific accumulation, boost stress tolerance, reduce anti-nutritional compounds, and facilitate the breeding of multifunctional crops (Figure 3).

CONCLUDING REMARKS AND FUTURE PERSPECTIVES

Membrane-localized transporters allocate numerous SMs within and between cells, move intermediates, and facilitate long-distance transport between organs. Although only a limited number of SM transporters have been functionally characterized in plants, recent evidence highlights their essential roles in directing metabolites to sites of synthesis and storage and in influencing overall plant metabolism and stress responses. These findings underscore the potential of transporters as targets for genetic engineering to enhance SM production, grain quality, and plant performance under environmental stresses. For transporter engineering to advance, functional validation and the development of effective modification strategies for SM transporters remain major challenges. This review focused on advances in understanding the transport of defense-related SMs, including alkaloids, GSLs, and selected bitter compounds. Systematic characterization of their short-distance and long-distance pathways, including SM exporters and importers on vacuoles, PMs, and vascular tissues, will deepen our understanding of source–sink relationships and metabolite flux. We also examined both successful and unsuccessful transporter manipulations to identify factors that influence outcomes. We emphasize the importance of selecting and targeting key long-distance transporters for the development of multifunctional crops with enhanced nutritional value, reduced toxicity, and maintained stress tolerance. Finally, we propose a workflow for exploring long-distance transport mechanisms of valuable metabolites to facilitate the discovery and functional validation of novel transporters. This knowledge and these strategies can be extended to other economically important plants, such as lupin, cassava, almond, and bitter melon, to reduce anti-nutritional compounds in edible parts while enhancing stress resilience.

Despite recent progress, significant challenges remain. A major limitation of genetic interventions is the unpredictability of outcomes due to incomplete characterization of transporter networks. Many transporters exhibit substrate promiscuity (transport of multiple, unrelated metabolites), functional redundancy (compensation by homologs), or pleiotropic effects (unintended changes in agronomic traits after manipulation). These complexities highlight the need for systematic approaches to resolve transporter specificity, regulation, and interactions. Integrating multi-omics data, for example transporter expression patterns coupled with metabolite flux maps, will be key to deciphering transport networks and regulatory hubs. Emerging platforms are poised to accelerate the discovery and characterization of SM transporters. High-throughput screening of transporters in yeast enables rapid functional testing of large gene sets (Groszmann et al., 2023). Mass spectrometry imaging provides spatial insights into transport routes (Buchberger et al., 2018). Single-cell RNA sequencing reveals cellular expression maps in complex tissues such as the xylem (Tung et al., 2023).

Additionally, AI and machine learning models offer predictive power for identifying transporter structures and substrates and for inferring co-expression patterns in gene networks (Theodoris et al., 2023; Kroll et al., 2024). Together, these platforms will support the construction of comprehensive transporter interaction networks that uncover redundancy, compensatory mechanisms, and source–sink dynamics governing SM distribution. Precision engineering approaches, particularly those based on CRISPR technology, should be further developed to enable targeted modulation of transporter activity across a wider range of species with specific spatial and temporal control, while minimizing off-target effects and improving metabolic control. By overcoming these challenges, engineering of SM transporters can move beyond incremental gains toward crops with tailored metabolite profiles that balance yield, defense, and nutritional quality. This will support modern breeding strategies and offer sustainable solutions to food security and environmental challenges.

FUNDING

This work was supported by the Australian Grains Research and Development Corporation (UMU2306-008RSX and UMU2404-007RTX).

ACKNOWLEDGMENTS

The author acknowledges financial support from a Murdoch International Postgraduate Scholarship at Murdoch University.

AUTHOR CONTRIBUTIONS

C.L. conceptualized the manuscript. C.X. drafted the manuscript and prepared all tables and figures. C.L., G.Z., and T.H. revised the manuscript, tables, and figures. All authors approved the final version of the manuscript for submission.

SUPPLEMENTAL INFORMATION

Supplemental information is available at *Plant Communications Online*.

Received: April 20, 2025
Revised: July 30, 2025
Accepted: September 24, 2025
Published: September 25, 2025

REFERENCES

Alagoz, Y., Gurkok, T., Zhang, B., and Unver, T. (2016). Manipulating the biosynthesis of bioactive compound alkaloids for next-generation metabolic engineering in opium poppy using CRISPR-Cas9 genome editing technology. Sci. Rep. **6**:30910.

Andersen, T.G., Nour-Eldin, H.H., Fuller, V.L., Olsen, C.E., Burow, M., and Halkier, B.A. (2013). Integration of biosynthesis and long-distance transport establish organ-specific glucosinolate profiles in vegetative *Arabidopsis*. Plant Cell **25**:3133–3145.

Anokhina, V., Kaminskaya, L., and Tsibulskaya, I. (2008). Lupine alkaloids: fungicidal effects. Mol. Appl. Genet. **8**:138–142.

Atkins, C.A., and Smith, P.M.C. (2007). Translocation in legumes: assimilates, nutrients, and signaling molecules. Plant Physiol. **144**:550–561.

Bai, Y., Liu, X., and Baldwin, I.T. (2024). Using synthetic biology to understand the function of plant specialized metabolites. Annu. Rev. Plant Biol. **75**:629–653.

Baldwin, I.T. (1989). Mechanism of damage-induced alkaloid production in wild tobacco. J. Chem. Ecol. **15**:1661–1680.

Baldwin, I.T. (2001). An ecologically motivated analysis of plant-herbivore interactions in native tobacco. Plant Physiol. **127**:1449–1458.

Buchberger, A.R., DeLaney, K., Johnson, J., and Li, L. (2018). Mass spectrometry imaging: a review of emerging advancements and future insights. Anal. Chem. **90**:240–265.

Chen, S., Petersen, B.L., Olsen, C.E., Schulz, A., and Halkier, B.A. (2001). Long-distance phloem transport of glucosinolates in *Arabidopsis*. Plant Physiol. **127**:194–201.

Cheng, L., Bucciarelli, B., Liu, J., Zinn, K., Miller, S., Patton-Vogt, J., Allan, D., Shen, J., and Vance, C.P. (2011). White lupin cluster root acclimation to phosphorus deficiency and root hair development involve unique glycerophosphodiester phosphodiesterases. Plant Physiol. **156**:1131–1148.

Chokkathukalam, A., Kim, D.H., Barrett, M.P., Breitling, R., and Creek, D.J. (2014). Stable isotope-labeling studies in metabolomics: new insights into structure and dynamics of metabolic networks. Bioanalysis **6**:511–524.

Clay, N.K., Adio, A.M., Denoux, C., Jander, G., and Ausubel, F.M. (2009). Glucosinolate metabolites required for an *Arabidopsis* innate immune response. Science **323**:95–101.

Dastmalchi, M., Chang, L., Chen, R., Yu, L., Chen, X., Hagel, J.M., and Facchini, P.J. (2019). Purine permease-type benzylisoquinoline alkaloid transporters in opium poppy. Plant Physiol. **181**:916–933.

Drewnowski, A., and Gomez-Carneros, C. (2000). Bitter taste, phytonutrients, and the consumer: a review. Am. J. Clin. Nutr. **72**:1424–1435.

Dwivedi, S.L., Reynolds, M.P., and Ortiz, R. (2021). Mitigating tradeoffs in plant breeding. iScience **24**:102965.

Freeman, B., and Beattie, G. (2008). An overview of plant defenses against pathogens and herbivores. Plant Health Instr **149**:2832–2833.

Gleadow, R.M., and Møller, B.L. (2014). Cyanogenic glycosides: synthesis, physiology, and phenotypic plasticity. Annu. Rev. Plant Biol. **65**:155–185.

Groszmann, M., De Rosa, A., Chen, W., Qiu, J., McGaughey, S.A., Byrt, C.S., and Evans, J.R. (2023). A high-throughput yeast approach to characterize aquaporin permeabilities: Profiling the *Arabidopsis* PIP aquaporin sub-family. Front. Plant Sci. **14**:1078220.

Halkier, B.A., and Xu, D. (2022). The ins and outs of transporters at plasma membrane and tonoplast in plant specialized metabolism. Nat. Prod. Rep. **39**:1483–1491.

Hang, J., Wu, B., Qiu, D., Yang, G., Fang, Z., and Zhang, M. (2024). OsNPF3.1, a nitrate, abscisic acid and gibberellin transporter gene, is essential for rice tillering and nitrogen utilization efficiency. J. Integr. Agric. **23**:1087–1104.

He, J., and Giusti, M.M. (2010). Anthocyanins: natural colorants with health-promoting properties. Annu. Rev. Food Sci. Technol. **1**:163–187.

He, Y., Yang, Z., Tang, M., Yang, Q.Y., Zhang, Y., and Liu, S. (2022). Enhancing canola breeding by editing a glucosinolate transporter gene lacking natural variation. Plant Physiol. **188**:1848–1851.

Hildreth, S.B., Gehman, E.A., Yang, H., Lu, R.H., Ritesh, K.C., Harich, K.C., Yu, S., Lin, J., Sandoe, J.L., Okumoto, S., et al. (2011). Tobacco nicotine uptake permease (NUP1) affects alkaloid metabolism. Proc. Natl. Acad. Sci. USA **108**:18179–18184.

Hunziker, P., Halkier, B.A., and Schulz, A. (2019). Arabidopsis glucosinolate storage cells transform into phloem fibres at late stages of development. J. Exp. Bot. **70**:4305–4317.

Hunziker, P., Lambertz, S.K., Weber, K., Crocoll, C., Halkier, B.A., and Schulz, A. (2021). Herbivore feeding preference corroborates optimal defense theory for specialized metabolites within plants. Proc. Natl. Acad. Sci. US **118**:e2111977118.

Ikezawa, N., Tanaka, M., Nagayoshi, M., Shinkyo, R., Sakaki, T., Inouye, K., and Sato, F. (2003). Molecular cloning and characterization of CYP719, a methylenedioxy bridge-forming enzyme that belongs to a novel P450 family, from cultured *Coptis japonica* cells. J. Biol. Chem. **278**:38557–38565.

Isah, T. (2019). Stress and defense responses in plant secondary metabolites production. Biol. Res. **52**:39.

Ishimaru, Y., Oikawa, T., Suzuki, T., Takeishi, S., Matsuura, H., Takahashi, K., Hamamoto, S., Uozumi, N., Shimizu, T., Seo, M., et al. (2017). GTR1 is a jasmonic acid and jasmonoyl-l-isoleucine transporter in *Arabidopsis thaliana*. Biosci. Biotechnol. Biochem. **81**:249–255.

Jørgensen, K., Bak, S., Busk, P.K., Sørensen, C., Olsen, C.E., Puonti-Kaerlas, J., and Møller, B.L. (2005). Cassava plants with a depleted cyanogenic glucoside content in leaves and tubers. Distribution of cyanogenic glucosides, their site of synthesis and transport, and blockage of the biosynthesis by RNA interference technology. Plant Physiol. **139**:363–374.

Jørgensen, M.E., Nour-Eldin, H.H., and Halkier, B.A. (2015). Transport of defense compounds from source to sink: lessons learned from glucosinolates. Trends Plant Sci. **20**:508–514.

Jørgensen, M.E., Xu, D., Crocoll, C., Ernst, H.A., Ramírez, D., Motawia, M.S., Olsen, C.E., Mirza, O., Nour-Eldin, H.H., and Halkier, B.A. (2017). Origin and evolution of transporter substrate specificity within the NPF family. eLife **6**:e19466.

Kato, K., Shitan, N., Shoji, T., and Hashimoto, T. (2015). Tobacco NUP1 transports both tobacco alkaloids and vitamin B6. Phytochemistry **113**:33–40.

Kazachkova, Y., Zemach, I., Panda, S., Bocobza, S., Vainer, A., Rogachev, I., Dong, Y., Ben-Dor, S., Veres, D., Kanstrup, C., et al. (2021). The GORKY glycoalkaloid transporter is indispensable for preventing tomato bitterness. Nat. Plants **7**:468–480.

Kroll, A., Niebuhr, N., Butler, G., and Lercher, M.J. (2024). SPOT: a machine learning model that predicts specific substrates for transport proteins. PLOS Biol. **22**:e2002807.

Kumar, P., Augustine, R., Singh, A.K., and Bisht, N.C. (2017). Feeding behaviour of generalist pests on: implication for manipulation of

glucosinolate biosynthesis pathway for enhanced resistance. Plant Cell Environ. **40**:2109–2120.

Larsen, B., Fuller, V.L., Pollier, J., Van Moerkercke, A., Schweizer, F., Payne, R., Colinas, M., O'Connor, S.E., Goossens, A., and Halkier, B.A. (2017). Identification of iridoid glucoside transporters in *Catharanthus roseus*. Plant Cell Physiol. **58**:1507–1518.

Lee, M.J., Pate, J.S., Harris, D.J., and Atkins, C.A. (2007). Synthesis, transport and accumulation of quinolizidine alkaloids in *Lupinus albus* L. and *L. angustifolius* L. J. Exp. Bot. **58**:935–946.

Lefèvre, F., and Boutry, M. (2018). Towards identification of the substrates of ATP-binding cassette transporters. Plant Physiol. **178**:18–39.

Lew, T.T.S., Park, M., Wang, Y., Gordiichuk, P., Yeap, W.C., Mohd rais, S.K., Kulaveerasingam, H., and Strano, M.S. (2020). Nanocarriers for transgene expression in pollen as a plant biotechnology tool. ACS Mater. Lett. **2**:1057–1066.

Liu, S., Huang, H., Yi, X., Zhang, Y., Yang, Q., Zhang, C., Fan, C., and Zhou, Y. (2020). Dissection of genetic architecture for glucosinolate accumulations in leaves and seeds of *Brassica napus* by genome-wide association study. Plant Biotechnol. J. **18**:1472–1484.

Liu, Y., Nour-Eldin, H.H., Zhang, L., Li, Z., Fernie, A.R., and Ren, M. (2023). Biotechnological detoxification: an unchanging source-sink balance strategy for crop improvement. Trends Plant Sci. **28**:135–138.

Liu, S., Grierson, D., and Xi, W. (2024). Biosynthesis, distribution, nutritional and organoleptic properties of bitter compounds in fruit and vegetables. Crit. Rev. Food Sci. Nutr. **64**:1934–1953.

Loza-Muller, L., Shitan, N., Yamada, Y., and Vázquez-Flota, F. (2021). AmABCB1, an alkaloid transporter from seeds of *Argemone mexicana L* (Papaveraceae). Planta **254**:122.

Lv, H., Li, J., Wu, Y., Garyali, S., and Wang, Y. (2016). Transporter and its engineering for secondary metabolites. Appl. Microbiol. Biotechnol. **100**:6119–6130.

Ma, X., Zhang, X., Liu, H., and Li, Z. (2020). Highly efficient DNA-free plant genome editing using virally delivered CRISPR/Cas9. Nat. Plants **6**:773–779.

Ma, K., Li, W., Zhu, G., Sun, S., Chi, H., Yin, Y., Diao, H., Xing, X.J., Guo, Z., Wang, L., et al. (2021). Functionalized PDA/DEX-PEI@HA nanoparticles combined with sleeping-beauty transposons for multistage targeted delivery of CRISPR/Cas9 gene. Biomed. Pharmacother. **142**:112061.

Ma, Y., Li, D., Zhong, Y., Wang, X., Li, L., Osbourn, A., Lucas, W.J., Huang, S., and Shang, Y. (2023). Vacuolar MATE/DTX protein-mediated cucurbitacin C transport is co-regulated with bitterness biosynthesis in cucumber. New Phytol. **238**:995–1003.

Madsen, S.R., Olsen, C.E., Nour-Eldin, H.H., and Halkier, B.A. (2014). Elucidating the role of transport processes in leaf glucosinolate distribution. Plant Physiol. **166**:1450–1462.

Mahroug, S., Burlat, V., and St-Pierre, B. (2007). Cellular and sub-cellular organisation of the monoterpenoid indole alkaloid pathway in *Catharanthus roseus*. Phytochem. Rev. **6**:363–381.

Mancinotti, D., Rodriguez, M.C., Frick, K.M., Dueholm, B., Jepsen, D. G., Agerbirk, N., and Geu-Flores, F. (2021). Development and application of a virus-induced gene silencing protocol for the study of gene function in narrow-leafed lupin. Plant Methods **17**:131.

Mann, A., Kumari, J., Kumar, R., Kumar, P., Pradhan, A.K., Pental, D., and Bisht, N.C. (2023). Targeted editing of multiple homologues of GTR1 and GTR2 genes provides the ideal low-seed, high-leaf glucosinolate oilseed mustard with uncompromised defense and yield. Plant Biotechnol. J. **21**:2182–2195.

Morita, M., Shitan, N., Sawada, K., Van Montagu, M.C.E., Inzé, D., Rischer, H., Goossens, A., Oksman-Caldentey, K.M., Moriyama, Y., and Yazaki, K. (2009). Vacuolar transport of nicotine is mediated by a multidrug and toxic compound extrusion (MATE) transporter in *Nicotiana tabacum*. Proc. Natl. Acad. Sci. USA **106**:2447–2452.

Nambiar, D.M., Kumari, J., Augustine, R., Kumar, P., Bajpai, P.K., and Bisht, N.C. (2021). GTR1 and GTR2 transporters differentially regulate tissue-specific glucosinolate contents and defense responses in the oilseed crop. Plant Cell Environ. **44**:2729–2743.

Nogia, P., and Pati, P.K. (2021). Plant secondary metabolite transporters: diversity, functionality, and their modulation. Front. Plant Sci. **12**:758202.

Nour-Eldin, H.H., and Halkier, B.A. (2009). Piecing together the transport pathway of aliphatic glucosinolates. Phytochem. Rev. **8**:53–67.

Nour-Eldin, H.H., and Halkier, B.A. (2013). The emerging field of transport engineering of plant specialized metabolites. Curr. Opin. Biotechnol. **24**:263–270.

Nour-Eldin, H.H., Andersen, T.G., Burow, M., Madsen, S.R., Jørgensen, M.E., Olsen, C.E., Dreyer, I., Hedrich, R., Geiger, D., and Halkier, B.A. (2012). NRT/PTR transporters are essential for translocation of glucosinolate defense compounds to seeds. Nature **488**:531–534.

Nour-Eldin, H.H., Madsen, S.R., Engelen, S., Jørgensen, M.E., Olsen, C.E., Andersen, J.S., Seynnaeve, D., Verhoye, T., Fulawka, R., Denolf, P., et al. (2017). Reduction of antinutritional glucosinolates in Brassica oilseeds by mutation of genes encoding transporters. Nat. Biotechnol. **35**:377–382.

Ogbonna, A.C., Braatz de Andrade, L.R., Rabbi, I.Y., Mueller, L.A., Jorge de Oliveira, E., and Bauchet, G.J. (2021). Large-scale genome-wide association study, using historical data, identifies conserved genetic architecture of cyanogenic glucoside content in cassava (*Manihot esculenta Crantz*) root. Plant J. **105**:754–770.

Otterbach, S.L., Yang, T., Kato, L., Janfelt, C., and Geu-Flores, F. (2019). Quinolizidine alkaloids are transported to seeds of bitter narrow-leafed lupin. J. Exp. Bot. **70**:5799–5808.

Payne, R.M.E., Xu, D., Foureau, E., Teto Carqueijeiro, M.I.S., Oudin, A., Bernonville, T.D.d., Novak, V., Burow, M., Olsen, C.E., Jones, D.M., et al. (2017). An NPF transporter exports a central monoterpene indole alkaloid intermediate from the vacuole. Nat. Plants **3**:16208.

Pigeaire, A., Abernethy, D., Smith, P.M., Simpson, K., Fletcher, N., Lu, C.-Y., Atkins, C.A., and Cornish, E. (1997). Transformation of a grain legume (*Lupinus angustifolius* L.) via *Agrobacterium tumefaciens*-mediated gene transfer to shoot apices. Mol. Breed. **3**:341–349.

Pike, S., Matthes, M.S., McSteen, P., and Gassmann, W. (2019). Using *Xenopus laevis oocytes* to functionally characterize plant transporters. Curr. Protoc. Plant Biol. **4**:e20087.

Qin, H., King, G.J., Borpatragohain, P., and Zou, J. (2023). Developing multifunctional crops by engineering Brassicaceae glucosinolate pathways. Plant Commun. **4**:100565.

Riaz, M., Khalid, R., Afzal, M., Anjum, F., Fatima, H., Zia, S., Rasool, G., Egbuna, C., Mtewa, A.G., Uche, C.Z., et al. (2023). Phytobioactive compounds as therapeutic agents for human diseases: A review. Food Sci. Nutr. **11**:2500–2529.

Ricigliano, V., Kumar, S., Kinison, S., Brooks, C., Nybo, S.E., Chappell, J., and Howarth, D.G. (2016). Regulation of sesquiterpenoid metabolism in recombinant and elicited *Valeriana officinalis* hairy roots. Phytochemistry **125**:43–53.

Roepke, J., Salim, V., Wu, M., Thamm, A.M.K., Murata, J., Ploss, K., Boland, W., and De Luca, V. (2010). Vinca drug components accumulate exclusively in leaf exudates of Madagascar periwinkle. Proc. Natl. Acad. Sci. USA **107**:15287–15292.

Sanden, N.C.H., Kanstrup, C., Crocoll, C., Schulz, A., Nour-Eldin, H.H., Halkier, B.A., and Xu, D. (2024). An UMAMIT-GTR transporter cascade controls glucosinolate seed loading in Arabidopsis. Nat. Plants **10**:172–179.

Sbabou, L., Bucciarelli, B., Miller, S., Liu, J., Berhada, F., Filali-Maltouf, A., Allan, D., and Vance, C. (2010). Molecular analysis of SCARECROW genes expressed in white lupin cluster roots. J. Exp. Bot. **61**:1351–1363.

Shang, Y., Ma, Y., Zhou, Y., Zhang, H., Duan, L., Chen, H., Zeng, J., Zhou, Q., Wang, S., Gu, W., et al. (2014). Biosynthesis, regulation, and domestication of bitterness in cucumber. Science **346**:1084–1088.

Shitan, N., and Yazaki, K. (2007). Accumulation and membrane transport of plant alkaloids. Curr. Pharm. Biotechnol. **8**:244–252.

Shitan, N., Bazin, I., Dan, K., Obata, K., Kigawa, K., Ueda, K., Sato, F., Forestier, C., and Yazaki, K. (2003). Involvement of CjMDR1, a plant multidrug-resistance-type ATP-binding cassette protein, in alkaloid transport in *Coptis japonica*. Proc. Natl. Acad. Sci. USA **100**:751–756.

Shitan, N., Kiuchi, F., Sato, F., Yazaki, K., and Yoshimatsu, K. (2005). Establishment of Rhizobium-mediated transformation of *Coptis japonica* and molecular analyses of transgenic plants. Plant Biotechnol. **22**:113–118.

Shitan, N., Dalmas, F., Dan, K., Kato, N., Ueda, K., Sato, F., Forestier, C., and Yazaki, K. (2013). Characterization of *Coptis japonica* CjABCB2, an ATP-binding cassette protein involved in alkaloid transport. Phytochemistry **91**:109–116.

Shitan, N., Kato, K., and Shoji, T. (2014a). Alkaloid transporters in plants. Plant Biotechnol. **31**:453–463.

Shitan, N., Minami, S., Morita, M., Hayashida, M., Ito, S., Takanashi, K., Omote, H., Moriyama, Y., Sugiyama, A., Goossens, A., et al. (2014b). Involvement of the leaf-specific Multidrug and Toxic Compound Extrusion (MATE) transporter Nt-JAT2 in vacuolar sequestration of nicotine in *Nicotiana tabacum*. PLoS One **9**:e108789.

Shitan, N. (2016). Secondary metabolites in plants: transport and self-tolerance mechanisms. Biosci. Biotechnol. Biochem. **80**:1283–1293.

Shoji, T., Inai, K., Yazaki, Y., Sato, Y., Takase, H., Shitan, N., Yazaki, K., Goto, Y., Toyooka, K., Matsuoka, K., et al. (2009). Multidrug and toxic compound extrusion-type transporters implicated in vacuolar sequestration of nicotine in tobacco roots. Plant Physiol. **149**:708–718.

Shoji, T., Winz, R., Iwase, T., Nakajima, K., Yamada, Y., and Hashimoto, T. (2002). Expression patterns of two tobacco isoflavone reductase-like genes and their possible roles in secondary metabolism in tobacco. Plant Mol. Biol **50**:427–440.

Sikora, P., Chawade, A., Larsson, M., Olsson, J., and Olsson, O. (2011). Mutagenesis as a tool in plant genetics, functional genomics, and breeding. Int. J. Plant Genom. **2011**:314829.

Srinivasan, P., and Smolke, C.D. (2021). Engineering cellular metabolite transport for biosynthesis of computationally predicted tropane alkaloid derivatives in yeast. Proc. Natl. Acad. Sci. USA **118**:e2104460118.

Steppuhn, A., Gase, K., Krock, B., Halitschke, R., and Baldwin, I.T. (2004). Nicotine's defensive function in nature. PLoS Biol. **2**:e217.

St-Pierre, B., Vazquez-Flota, F.A., and De Luca, V. (1999). Multicellular compartmentation of *Catharanthus roseus* alkaloid biosynthesis predicts intercellular translocation of a pathway intermediate. Plant Cell **11**:887–900.

Takanashi, K., Yamada, Y., Sasaki, T., Yamamoto, Y., Sato, F., and Yazaki, K. (2017). A multidrug and toxic compound extrusion transporter mediates berberine accumulation into vacuoles in *Coptis japonica*. Phytochemistry **138**:76–82.

Tan, Z., Xie, Z., Dai, L., Zhang, Y., Zhao, H., Tang, S., Wan, L., Yao, X., Guo, L., and Hong, D. (2022). Genome- and transcriptome-wide association studies reveal the genetic basis and the breeding history of seed glucosinolate content in *Brassica napus*. Plant Biotechnol. J. **20**:211–225.

Theodoris, C.V., Xiao, L., Chopra, A., Chaffin, M.D., Al Sayed, Z.R., Hill, M.C., Mantineo, H., Brydon, E.M., Zeng, Z., Liu, X.S., et al. (2023). Transfer learning enables predictions in network biology. Nature **618**:616–624.

Tung, C.C., Kuo, S.C., Yang, C.L., Yu, J.H., Huang, C.E., Liou, P.C., Sun, Y.H., Shuai, P., Su, J.C., Ku, C., et al. (2023). Single-cell transcriptomics unveils xylem cell development and evolution. Genome Biol. **24**:3.

Uhde-Stone, C., Liu, J., Zinn, K.E., Allan, D.L., and Vance, C.P. (2005). Transgenic proteoid roots of white lupin: a vehicle for characterizing and silencing root genes involved in adaptation to P stress. Plant J. **44**:840–853.

Verma, P., Mathur, A.K., Srivastava, A., and Mathur, A. (2012). Emerging trends in research on spatial and temporal organization of terpenoid indole alkaloid pathway in *Catharanthus roseus*: a literature update. Protoplasma **249**:255–268.

Wang, Y., Yang, B., Zhang, M., Jia, S., and Yu, F. (2019). Application of transport engineering to promote catharanthine production in *Catharanthus roseus* hairy roots. Plant Cell Tissue Organ Cult. **139**:523–530.

Wang, R., Liu, Y., Xu, S., Li, J., Zhou, J., and Wang, R. (2021). An ATP-binding cassette transporter, LaABCB11, contributes to alkaloid transport in *Lycoris aurea*. Int. J. Mol. Sci. **22**:11458.

Wang, L.H., Tan, D.H., Zhong, X.S., Jia, M.Q., Ke, X., Zhang, Y.M., Cui, T., and Shi, L. (2024). Review on toxicology and activity of tomato glycoalkaloids in immature tomatoes. Food Chem. **447**:138937.

Watson, A., Ghosh, S., Williams, M.J., Cuddy, W.S., Simmonds, J., Rey, M.D., Asyraf Md Hatta, M., Hinchliffe, A., Steed, A., Reynolds, D., et al. (2018). Speed breeding is a powerful tool to accelerate crop research and breeding. Nat. Plants **4**:23–29.

Wink, M. (2010). Mode of action and toxicology of plant toxins and poisonous plants. Wirbeltier for schung in der Kulturl and schaft **421**:93–112.

Wu, T., Kerbler, S.M., Fernie, A.R., and Zhang, Y. (2021). Plant cell cultures as heterologous bio-factories for secondary metabolite production. Plant Commun. **2**:100235.

Xu, D., Hanschen, F.S., Witzel, K., Nintemann, S.J., Nour-Eldin, H.H., Schreiner, M., and Halkier, B.A. (2017). Rhizosecretion of stele-synthesized glucosinolates and their catabolites requires GTR-mediated import in *Arabidopsis*. J. Exp. Bot. **68**:3205–3214.

Xu, D., Hunziker, P., Koroleva, O., Blennow, A., Crocoll, C., Schulz, A., Nour-Eldin, H.H., and Halkier, B.A. (2019). GTR-mediated radial import directs accumulation of defensive glucosinolates to sulfur-rich cells in the phloem cap of *Arabidopsis* inflorescence stem. Mol. Plant **12**:1474–1484.

Xu, D., Sanden, N.C.H., Hansen, L.L., Belew, Z.M., Madsen, S.R., Meyer, L., Jørgensen, M.E., Hunziker, P., Veres, D., Crocoll, C., et al. (2023). Export of defensive glucosinolates is key for their accumulation in seeds. Nature **617**:132–138.

Yamada, Y., Nakagawa, A., Sato, F., Minami, H., and Shitan, N. (2022). Transport engineering using tobacco transporter NtJAT1 enhances alkaloid production in *Escherichia coli*. Biosci. Biotechnol. Biochem. **86**:865–869.

Yazaki, K., Shitan, N., Takamatsu, H., Ueda, K., and Sato, F. (2001). A novel *Coptis japonica* multidrug-resistant protein preferentially

expressed in the alkaloid-accumulating rhizome. J. Exp. Bot. **52**:877–879.

Yazaki, K., Sugiyama, A., Morita, M., and Shitan, N. (2008). Secondary transport as an efficient membrane transport mechanism for plant secondary metabolites. Phytochem. Rev. **7**:513–524.

Yu, F., and De Luca, V. (2013). ATP-binding cassette transporter controls leaf surface secretion of anticancer drug components in *Catharanthus roseus*. Proc. Natl. Acad. Sci. USA **110**:15830–15835.

Zhang, D., Zhang, Z., Unver, T., and Zhang, B. (2021). CRISPR/Cas: A powerful tool for gene function study and crop improvement. J. Adv. Res. **29**:207–221.

Zhang, Y., Wei, K., Guo, L., Lei, Y., Cheng, H., Chen, C., and Wang, L. (2022). Functional identification of purine permeases reveals their roles in caffeine transport in tea plants (*Camellia sinensis*). Front. Plant Sci. **13**:1033316.

Zhao, X., Meng, Z., Wang, Y., Chen, W., Sun, C., Cui, B., Cui, J., Yu, M., Zeng, Z., Guo, S., et al. (2017). Pollen magnetofection for genetic modification with magnetic nanoparticles as gene carriers. Nat. Plants **3**:956–964.

Zhao, J. (2015). Flavonoid transport mechanisms: how to go, and with whom. Trends Plant Sci. **20**:576–585.

Plant Communications, Volume *6*

Supplemental information

Transport of secondary metabolites in plants: Mechanistic insights and transporter engineering for crop improvement

Chunsheng Xiao, Gaofeng Zhou, Tianhua He, and Chengdao Li

Supplementary Table

TableS1. List of abbreviations and their definitions.

Abbreviation	Full Term	Description
ABC	ATP-binding Cassette	Membrane proteins that transport numerous substrates across membranes, including secondary metabolites, via ATP hydrolysis.
CuC	Cucurbitacin C	Triterpenoid compound found in cucurbit plants (e.g., cucumber, melon) that has strong antifeedant properties and contributes to plant defense.
GLSs	Glucosinolates	Sulfur- and nitrogen-containing secondary metabolites that primarily present in *Brassicaceae* plants and affect crop flavor, nutrition and defense ability
GTRs	Glucosinolate transporters	Specific transport proteins belong to NPF family that mediate the long-distance translocation of glucosinolates.
MATE	Multidrug and toxic compound extrusion	Transport proteins that mediate the efflux of secondary metabolites using proton gradients.
MIAs	Monoterpene indole alkaloids	Secondary metabolites derived from tryptamine and monoterpenes, known for their medicinal properties and commonly found in Catharanthus roseus.
NPF	Nitrate peptide family	Transporters family responsible for transporting nitrate and peptides, plant hormones and secondary metabolites.
PUP	Purine uptake permeases	Membrane transporters originally identified for purine transport and also mediate uptake of secondary metabolites.
SGAs	Steroidal glycoalkaloids	Secondary metabolites containing nitrogen found in Solanaceae species (e.g., tomato and potato).
SMs	Secondary metabolites	Organic compounds produced by plants that play critical roles in defense, signaling, and environmental adaptation.
UMAMITs	Usually multi-amino acids move in and out transporters	Transport family participate in amino acid exchange between cells and play a role in transporting secondary metabolites as well.
